# Modeling the Diversity of Epithelial Ovarian Cancer through Ten Novel Well Characterized Cell Lines Covering Multiple Subtypes of the Disease

**DOI:** 10.3390/cancers12082222

**Published:** 2020-08-08

**Authors:** Skye Alexandre Sauriol, Kayla Simeone, Lise Portelance, Liliane Meunier, Kim Leclerc-Desaulniers, Manon de Ladurantaye, Meriem Chergui, Jennifer Kendall-Dupont, Kurosh Rahimi, Euridice Carmona, Diane M. Provencher, Anne-Marie Mes-Masson

**Affiliations:** 1 Centre de recherche du Centre hospitalier de l’Université de Montréal (CRCHUM), Montreal, QC H2X 0A9, Canada; alex.sauriol@outlook.com (S.A.S.); kayla.simeone@outlook.com (K.S.); lise.portelance.chum@ssss.gouv.qc.ca (L.P.); liliane.meunier.chum@ssss.gouv.qc.ca (L.M.); kim.leclerc-desaulniers.chum@ssss.gouv.qc.ca (K.L.-D.); manon.deladurantaye.chum@ssss.gouv.qc.ca (M.d.L.); meriemc19@outlook.com (M.C.); jennifer.kendall-dupont.chum@ssss.gouv.qc.ca (J.K.-D.); euridice.carmona.chum@ssss.gouv.qc.ca (E.C.); diane.provencher.chum@ssss.gouv.qc.ca (D.M.P.); 2 Institut du cancer de Montréal, Montreal, QC H2X 0A9, Canada; 3 Department of Pathology, Centre hospitalier de l’Université de Montréal (CHUM), Montreal, QC H2X 3E4, Canada; kurosh.rahimi.chum@ssss.gouv.qc.ca; 4 Division of Gynecologic Oncology, Université de Montréal, Montreal, QC H3C 3J7, Canada; 5 Department of Medicine, Université de Montréal, Montreal, QC H3T 1J4, Canada

**Keywords:** epithelial ovarian cancer, high-grade serous, mucinous, clear cell, cell lines, xenograft, carboplatin, mutation profile, gene expression, biomarkers

## Abstract

Cancer cell lines are amongst the most important pre-clinical models. In the context of epithelial ovarian cancer, a highly heterogeneous disease with diverse subtypes, it is paramount to study a wide panel of models in order to draw a representative picture of the disease. As this lethal gynaecological malignancy has seen little improvement in overall survival in the last decade, it is all the more pressing to support future research with robust and diverse study models. Here, we describe ten novel spontaneously immortalized patient-derived ovarian cancer cell lines, detailing their respective mutational profiles and gene/biomarker expression patterns, as well as their in vitro and in vivo growth characteristics. Eight of the cell lines were classified as high-grade serous, while two were determined to be of the rarer mucinous and clear cell subtypes, respectively. Each of the ten cell lines presents a panel of characteristics reflective of diverse clinically relevant phenomena, including chemotherapeutic resistance, metastatic potential, and subtype-associated mutations and gene/protein expression profiles. Importantly, four cell lines formed subcutaneous tumors in mice, a key characteristic for pre-clinical drug testing. Our work thus contributes significantly to the available models for the study of ovarian cancer, supplying additional tools to better understand this complex disease.

## 1. Introduction

Ovarian cancer is the most lethal gynaecological cancer, and fifth leading cause of malignancy-related deaths in North American women [1]. Ovarian cancer is most often detected at a later stage, when the patient shows distant metastases, largely owing to the asymptomatic nature of early disease, followed by lack of specific symptoms at later stages [2,3]. The 5-year survival rate of ovarian cancer at its late stage is 29% [2,3]. Moreover, ovarian cancer is a heterogeneous group of diseases, which can be classified based on various factors including site of origin, genomic stability, and prognosis, with the most common form being epithelial ovarian cancer (EOC), representing 90% of all cases [4]. EOC is further classified into subtypes with differences in morphology, etiology, pathogenesis, pathology, molecular biology, and prognosis. High-grade serous carcinoma (HGSC) is diagnosed in 70% of the cases. Less common subtypes include mucinous carcinomas (MCs), low-grade serous carcinomas (LGSCs), clear-cell carcinomas (CCCs), and endometrioid carcinomas (ECs), accounting for 2–3%, <5%, 5–10%, and 10% of cases of EOC, respectively [5,6].

The most common risk factor for EOC is a family history of ovarian cancer. An estimated 25% of all ovarian cancer cases could be due to inherited genetic mutations, the most frequent being a germline *BRCA1* or *BRCA2* mutation but also, to a lesser degree, germline mutations in *RAD51C*, *RAD51D*, *PALB2*, and *FANCI*, with a higher incidence in the HGSC subtype [7,8,9,10,11]. It has also been shown that germline mutations in the DNA mismatch repair genes, known as Lynch syndrome, are an important risk factor for the EC and CCC subtypes of EOC [12]. Furthermore, EOC tumors usually present somatic mutational profiles that are characteristic to each subtype. Notably, in addition to a high proportion of germline *BRCA1/2* mutations, HGSC tumors harbor a virtually ubiquitous somatic *TP53* mutation (>95%), as well as less commonly mutated genes, including *CSMD3*, *NF1*, *CDK12*, and *FAT3* [13,14]. LGSCs, on the other hand, are almost never *TP53*-mutated, and instead show high-frequency mutually exclusive mutations in *KRAS*, *BRAF*, *NRAS*, or *ERBB2* [15,16,17]. CCC tumors often harbor *PIK3CA*, *ARID1A*, and *KRAS* mutations, amongst others, as well as a frequent loss of *PTEN* expression [15,16,18,19]. MC tumors are characterized by a high frequency of *KRAS* mutation, in addition to *TP53*, *BRAF*, and *CDKN2A* alterations [15,16,20]. EC tumors have a high frequency of *ARID1A* mutations, as well as moderately frequent mutations of *PIK3CA*, *CTNNB1*, and *PTEN*, usually presented as loss of expression or loss of heterozygosity, with some tumors rarely harboring a *TP53* mutation [15,16,21]. Despite being different diseases, the multiple subtypes of EOC are treated with the same standard front-line therapy: primary debulking surgery, and chemotherapy as an adjuvant or neo-adjuvant setting. Standard chemotherapy for front-line therapy in EOC is a combination of the DNA cross-linking compound carboplatin and the microtubule-stabilizing drug paclitaxel [2,3,22,23,24]. Although initial response rates are high (80%), the disease eventually recurs in 4 out of 5 patients, who then develop chemoresistance [25,26]. Over the past 30 years, advances in surgery, chemotherapy, targeted therapy agents (such as bevacizumab and poly (ADP-ribose) polymerase (PARP) inhibitors) and other evolving therapies (such as immunotherapy) have improved the 5-year progression-free survival rate but have had little impact on overall patient survival [2,3,4,27,28,29,30,31], underscoring the need for the development of new clinical tools for the management of EOC patients. Therefore, reliable and specific pre-clinical models are needed for the emergence of significant novel EOC drugs in clinical trials.

In this context, patient tumor-derived cell lines are efficient, inexpensive, and easy to maintain, and thus make for an attractive human pre-clinical cancer model [32,33]. Ovarian cancer being highly heterogeneous, a larger number of models need to be studied to cover the heterogeneity seen in the clinic, and a wide variety of cell lines is necessary to get a representative and accurate picture of the disease. Currently, available EOC cell lines are limited in number and characterization, especially when it comes to less common subtypes [15]. In addition, the relevance of the most commonly used cell lines as HGSC models has been called into question, suggesting that these might not be representative of the disease that they model [34,35], highlighting the need for more diversity of HGSC cell lines. We previously derived and characterized 22 EOC cell lines (from 16 patients) [36,37,38,39], with 19 being HGSC, as well as one of each LGSC, EC, and CCC, which were used to better understand the biology of ovarian cancer.

Here, we present 10 novel patient-derived EOC cell lines (from 10 different patients) and their generation, and describe their in vitro and in vivo growth characteristics, molecular biology, genetic mutation profiles, and histopathology. Eight of these cell lines were identified as HGSC cells, and two were found to be of the MC and CCC subtypes, respectively. All of the HGSC cell lines were *TP53*-mutated, whereas the MC and CCC cell lines were *TP53* wild-type but harbored KRAS mutations specific to their respective subtypes. Importantly, four of these cell lines form xenograft tumors in mice, a key characteristic for pre-clinical drug testing. These 10 new cell lines contribute significantly to the EOC cell lines available for research by their diversity and molecular characteristics.

## 2. Results

### 2.1. Patient Tumor-Derived EOC Cell Lines

The cell lines were derived from the samples of 10 patients (2085, 2414, 2835, 2881, 2929, 2978, 3121, 3291, 3331, and 3392) treated between 2001 and 2010, whose clinical characteristics are described in Table 1; Table 2 (and Table S1 and Figure S1). Of note, all patients were diagnosed with late stage (IIIC) disease between 42 and 77 years of age and died of disease progression between 2 to 86 months after diagnosis. They were treated with platinum-based chemotherapy, in combination with paclitaxel (or cyclophosphamide, in the case of 2414). Most patients (6/10) showed complete initial response to chemotherapy for at least one month after the end of the last cycle, as defined by the Gynecological Cancer Intergroup (CGIC) [40,41]. However, when platinum sensitivity was evaluated based on previously described consensus [2,3,40,42], the majority of patients (7/10) were categorized as resistant (recurrence at <6 months from the end of first-line treatment) or refractory (recurrence during first-line treatment) (Table 2). Patients 2881 and 3121 received neo-adjuvant chemotherapy, whereas patient 3331 did not undergo cytoreductive surgery. More than half (5/9) of the patients who did receive cytoreductive surgery were free of residual disease (Table 2). Because all patients were treated prior to 2010, none of them received PARP inhibitors, which have recently become part of the therapeutic armamentarium for this disease [43].

None of the patients harbored a somatic BRCA mutation. Patients 2085 and 2881 had a family history of breast cancer and malignant neoplasm of the urinary tract, respectively, whereas patients 2929 and 3392 had a previous personal history of colorectal cancer of the sigmoid junction and breast cancer, respectively. Histopathology indicated that 7 of the 10 primary tumors were of the HGSC subtype, one was of the MC subtype (2414), and one was of the CCC subtype (3392). As patient 3331 did not undergo surgery, histopathology was not possible, but cytology confirmed that the patient’s tumor was an adenocarcinoma (AC).

Sampling that resulted in the described cell lines for each of the 10 patients was done between 2004 and 2007. This sampling coincided with initial debulking surgery in the case of 6 of the patients (2929, 2978, 3121, 3291, 2414, 3392) or was done at a later point during laparotomy (2835, 2881) or ascites collection (2085, 3331) (Table 2 and Figure S1). Four of the patients (2929, 2978, 3291, 2414) were chemonaïve at the time of sampling. Patient 3392 was naïve to ovarian cancer treatment but received prior chemotherapy for breast cancer. The samples that served to derive the cell lines were either ascites (OV_) or solid tumor tissue of the ovary (TOV_). TOV cell lines were derived from samples of the right ovary (_D) or an unspecified ovary (TOV2414), or from metastases at the omentum (_EP) (Table 2).

**Figure 1:**
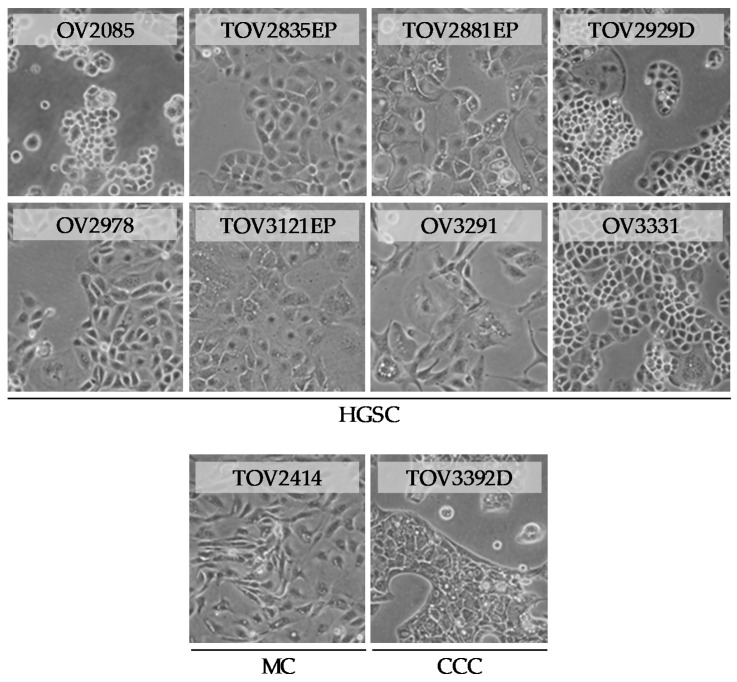
Morphology of 10 new patient-derived epithelial ovarian cancer (EOC) cell lines. Shown are brightfield microscopy pictures of each cell line, between passages 60 and 75, except in the case of OV3291, represented at passage 33. At these passages, cells exhibited uniform morphology, and cell lines were devoid of fibroblast-shaped cells. All pictures were taken at a magnification of 100×.

Nine of the ten cell lines displayed homogenous morphology after 60 passages, whereas one of the cell lines, OV3291, exhibited slowed growth after 50 passages and reached growth arrest before reaching 60 passages. This cell line was thus tested after reaching 30 passages, but before reaching passage 45. For the most part, the morphology of the cell lines exhibited the characteristic flattened cobblestone-like appearance of epithelial cells (Figure 1). Two of the cell lines, OV3291 and TOV2414, showed a more stretched spindle-like morphology, whereas OV2085 displayed small clusters of loosely attached round cells, which grew perpendicularly to the culture plate. TOV3392D, which had a characteristic flat epithelial morphology, grew in very dense clusters, gaining little confluence despite the increase in cell count (Figure 1).

### 2.2. Characteristic Subtype-Specific EOC Mutation and Gene Expression Profiles

Subtypes of EOC are known to harbor certain specific high-frequency mutations [13,15,16,18,20,21,44]. Using whole exome sequencing (WES) and selective mutation analyses, we surveyed the various subtype-specific recurrent mutations described by TCGA (for HGSC) and by various reports (for EC, MC, and CCC) [13,15,16,18,20,21]. All analyzed cell lines that were described as HGSC, as well as OV3331, harbored a *TP53* mutation; most (5/8) were missense mutations with amino acid substitutions (Table 3). The remaining *TP53* mutations are two frameshift mutations due to a nucleotide deletion (TOV3121EP, OV3331) and a splicing variant mutation (OV2978). All of these *TP53* mutations are described in the IARC TP53 database [45] and code for non-functional variants. The two non-HGSC cell lines TOV2414 and TOV3392D, on the other hand, did not harbour any *TP53* mutations (Table 3). These results correspond to what is expected of the analyzed subtypes [13,46,47,48].

Of the other described rare but recurrent mutations in HGSC [13], we identified a splicing mutation of *CDK12* in OV3291, a missense mutation of *FAT3* in TOV2881EP, and a missense mutation of *CSMD3* in TOV3121EP. Both missense mutations lead to amino acid substitutions. No mutations were detected in any other recurrent mutated genes in HGSC, including *BRCA1*, *BRCA2, NF1*, *GABRA6*, and *RB1* (Table 3). We identified recurrent mutations in *KRAS* in both the MC (TOV2414) and CCC (TOV3392D) cell lines analyzed but did not detect mutations in any of the other prevalently mutated genes in these subtypes, including *PIK3CA*, *ARID1A*, *BRAF*, *PTEN, CTNNB1, PPP2R1A,* and *NRAS* (Table 3) [16,19,49,50,51,52]. Of note, mutations identified in the OV2978 (*TP53*) and OV3291 (*TP53* and *CDK12*) cell lines (Table 3) are identical to their matched tumor cell lines (TOV2978G and TOV3291G), described in our previous publication [39].

**Figure 2:**
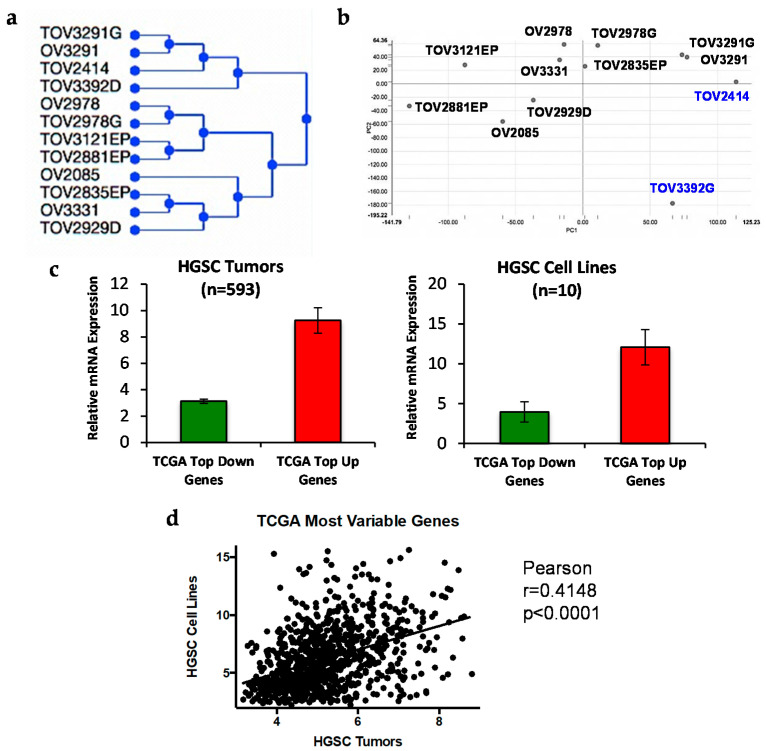
Gene expression analysis of our EOC cell lines, and comparison to tumor samples from the TCGA high-grade serous carcinoma (HGSC) dataset. (**a**,**b**) The complete normalized gene expression data for each of the 12 analyzed cell lines (10 from this work plus the two matched tumor cell lines from patients 2978 and 3291) was subjected to (**a**) unsupervised hierarchical clustering and (**b**) principal component analysis (PCA) using the TM4 MeV software. (**c**) Expression of the 1000 most up- or downregulated genes in HGSC tumors from the TCGA cohort (**left**) was verified in 10 of our HGSC cell lines (8 from this work plus the two matched TOV2978G and TOV3291G cell lines) (**right**) and plotted as bar graphs (mean ± SEM). (**d**) Expression of the 1000 most variably expressed genes in the TCGA HGSC cohort was verified in the 10 HGSC cell lines mentioned in (**c**), and Pearson correlation analysis was performed.

Cell lines were also characterized at the transcriptome level by gene expression microarray. Non-supervised hierarchical clustering demonstrated that seven of the eight HGSC cell lines clustered together (the exception being OV3291) and that the MC and CCC cell lines grouped in a separate cluster, confirming the distinct molecular identity of the derived cell lines (Figure 2a). Cell lines OV2978 and OV3291 clustered together with their matched cell lines, TOV2978G and TOV3291G. Additional clustering information was obtained by principal component analysis (PCA) (Figure 2b), which illustrates separation of the non-HGSC cell lines from the rest.

To further demonstrate the clinical relevance of our established cell lines, we compared the gene expression profiles of our eight HGSC cell lines, as well as the two matched TOV2978G and TOV3291G cell lines, with that of 593 HGSC tumors from the TCGA dataset publicly available at the UCSC Xena platform [53]. We selected the top 1000 up- or downregulated genes in the TCGA tumor samples and verified their expression in our HGSC cell lines. Our results (Figure 2c) show a striking similarity in the expression of these genes between our cell lines and the tumor samples. Then, following a previously described procedure [54], we selected the 1000 most variably expressed genes in the TCGA dataset and obtained a significant positive correlation (Pearson correlation analysis) with our cell lines (r = 0.4148, *p* < 0.0001) (Figure 2d). These results, together with our mutational profiling data, strongly suggest that our cell lines retain the molecular characteristics of the ovarian cancer disease.

### 2.3. Comparison of Subtype-Specific Biomarkers in Tumor Tissue and Cell Lines

**Figure 3:**
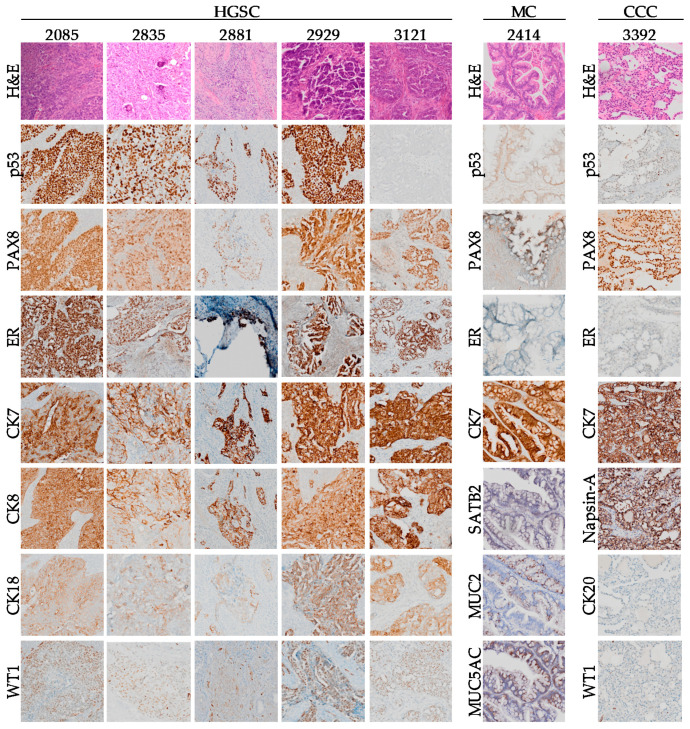
Immunohistochemistry staining for ovarian cancer markers. Shown are immunohistochemistry (IHC) staining of the tumor of origin from which each cell line was derived, separated by subtype. Each tumor was tested for relevant biomarkers for its respective subtype.

Due to the selection pressure inherent in establishing immortalized cell lines, it is important to verify that the cell line obtained reflects the EOC subtype of the original tumor. Therefore, we performed specific protein biomarker analyses of tumor tissue samples and cell lines by immunohistochemistry (IHC) and Western blot (WB), respectively. The tumor tissue of origin of the corresponding patient was subjected to a hematoxylin and eosin (H&E) staining, followed by IHC stains for various described epithelial ovarian cancer biomarkers [55] (Figure 3 and Figures S2 and S3). The representative epithelial malignant region characteristic of each EOC subtype [56,57] was selected based on morphology by H&E stain by a gynaecologic-oncology pathologist (KR), and the same region was used for each biomarker tested by IHC.

Previous work has shown the H&E and IHC staining of the tumor of origin for patients 3291 and 2978 [39], and an extract of this published data is shown in Figure S3. Patient 3331, on the other hand, did not receive surgery, thus preventing us from testing the tumor of origin of its corresponding cell line.

**Figure 4:**
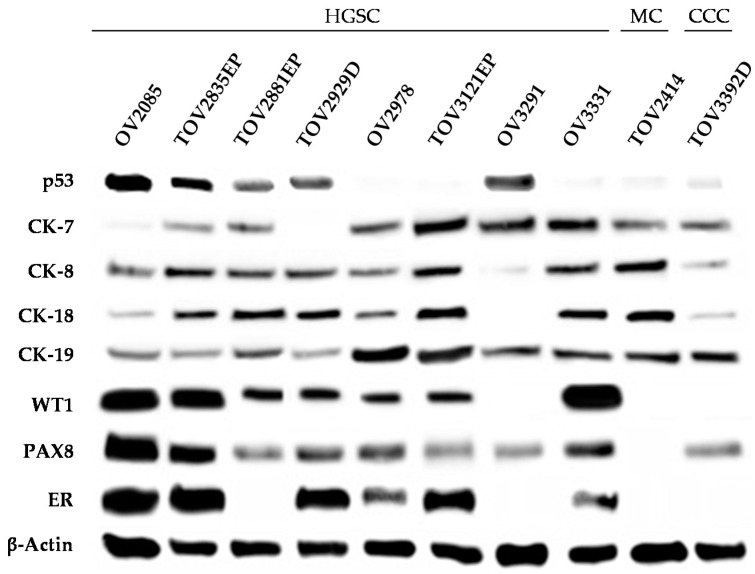
Protein expression of EOC subtype-specific markers in tumor cell lines. Detection of characteristic subtype-specific EOC markers (p53; cytokeratins 7, 8, 18, and 19; WT1; PAX8; and ER) of whole cell lysates of each cell line. β-Actin was used as control (*n* = 3).

Expression of cytokeratins (CK7, CK8, CK18, and CK19) confirmed the epithelial origin of the patient tumors (Figure 3, Figures S2 and S3a) and associated cell lines (Figure 4, Figures S3b and S4). The expression of specific biomarkers was then investigated to confirm the subtype of each patient tumor and derived cell line. In the case of HGSC tumors, *TP53* is mutated in more than 95% of cases, which is reflected at the expression level either by robust overexpression (missense mutation) or complete absence (null mutation) of the p53 protein [55,56,58]. As expected, p53 is strongly overexpressed in all HGSC tumors and cell lines harboring missense *TP53* mutations (Figure 3; Figure 4, Table 3), but is absent in 3121 and 3331 that harbor frameshift null mutations, as well as in 2978 that harbors a splicing variant mutation. On the other hand, tissue from patients 2414 and 3392 showed intermediate levels of p53, typical of the wild-type *TP53* genotype observed in their corresponding cell lines (Figure 3 and Figure 4, Table 3). To confirm that the frameshift and splice *TP53* mutations identified in our cell lines would not code for a truncated p53 protein that would not be recognized by the antibody used, we verified the *TP53* mRNA expression of our cell lines using our microarray data. Our results showed that the three cell lines harboring frameshift or splice mutations have absent to very low mRNA levels of *TP53*, corroborating the absence of protein expression on the Western blot (Figure S5a). On the other hand, wild-type *TP53* or missense mutations showed high levels of mRNA expression. Furthermore, we confirmed that in all the *TP53* mutated cell lines, p53 is not functional, as evidenced by absent or very low mRNA levels of *CDKN1A* (p21), a known p53 target [59,60], as opposed to high levels of *CDKN1A* in the wild-type *TP53* cell lines (Figure S5b).

**Figure 5:**
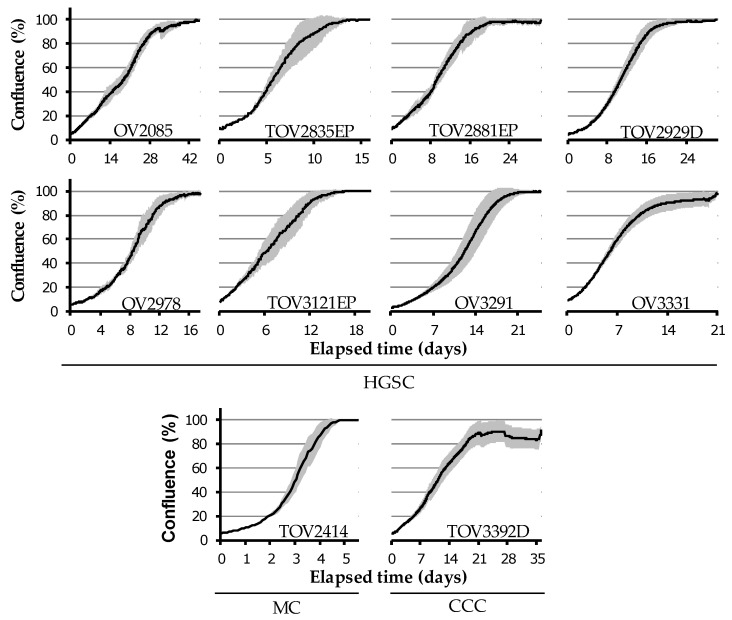
Confluence-based proliferation curves by live cell imaging. Cell proliferation of each cell line was determined by measuring confluence every 2 h. Initial values of confluence were between 5 and 10%, and cells were left to proliferate until confluence reached approximately 100%. Grey zones represent SEM (*n* = 3).

Amongst the other markers for HGSC, PAX8 is expressed across all EOC tumors, whereas Wilms tumor 1 (WT1) is solely expressed in the HGSC tumors (Figure 3 Figure S3). However, the ascites-derived cell line OV3291 did not express WT1 (Figure 4), despite its associated tumor of origin and corresponding tumor cell line TOV3921G expressing this protein (Figure S5) [39]. A common origin for these two matched cell lines was demonstrated by the presence of identical *TP53* and *CDK12* mutations, as well as by their proximal gene expression clustering (Table 3, Figure 5a). Intriguingly, OV3291 cells expressed *WT1* mRNA, and the expression of the WT1 protein was not increased by inhibition of the proteasome (Figure S6), ruling out stability as the mechanism of regulation responsible for the absence of WT1 expression. Further investigation is warranted to fully understand this phenomenon.

The Western blot analysis of the TOV2414 cell line, on the other hand, showed a lack of PAX8 expression (Figure 4). This discrepancy between tissue and cell line can be explained by the focal localization of this protein in the tumor tissue, which would have been lost by random selection during the establishment of the TOV2414 cell line.

Primary ovarian MC and colorectal metastases to the ovary overlap on many commonalities, making them difficult to differentiate [57,61]. Most notably, absence of SATB2 expression, focal expression of PAX-8, positive expression of MUC5AC and MUC2, and to a lesser extent, positive expression of CK20, strongly suggest that the tumor from patient 2414 is of the mucinous subtype of ovarian origin, rather than a metastasis from the colon, the appendix or the endometrium (Figure 3 and Figure S2) [62,63,64]. This is in spite of positive expression of CDX2 (Figure S2), which is more frequently positive in colorectal MC than ovarian MC (50% versus 38.3%, respectively) [65].

Ovarian CCC, on the other hand, is often confused with metastatic endometrial or renal clear cell carcinoma (RCCC), due to overlapping characteristics [66,67,68,69]. The tumor of origin of patient 3392 tests positive for Napsin A, and tests negative for estrogen receptor (ER), progesterone receptor (PR), WT1, and ARID1A (Figure 3 and Figure 4), suggesting that the tumor was not of endometrial origin [55,66,67]. In addition, positive staining for CK7 and p16 (focal and patchy), as well as negative staining for CK20 (Figure 3 and Figure 4 and Figure S2), set 3392 apart from RCCC [68,69,70]. Taken together, these results are strong evidence suggesting that the 3392 tumor and its corresponding cell line are of the CCC subtype and of ovarian origin.

In addition, we showed that all HGSC cell lines, but not the MC or CCC, express high levels of the *CDKN2A* mRNA (p16 protein) (Figure S5c), which is a distinct feature of HGSC tumors [71,72].

Tumor expression of ER and PR was shown to help distinguish the different subtypes of EOC [55,73]. Our results show that most analyzed HGSC tumors (2085, 2835, 2929, 3121) presented high levels of ER expression and absent or patchy expression of PR, whereas expression of both ER and PR in the MC (2414) and CCC (3392) tumors was absent (Figure 2 and Figure S3), as expected for these subtypes [55,73]. Expression of ER in each tumor’s corresponding cell line was confirmed by WB, which correlated with expression in the tumor of origin (Figure 3). Protein expression of human epidermal growth factor receptor-2 (HER2) is associated with malignant transformation and is overexpressed in serous EOC subtypes in 10–20% of cases [74,75]. HER2 was shown to be expressed in the tumor of origin of patient 3291 (Figure S3a) [39], but we did not detect expression of this protein in any of the other solid tumors tested (Figure S2).

Collectively, our protein biomarker analysis together with our gene expression and mutational profiling demonstrate the suitability of our cell lines as pre-clinical models to specific EOC subtypes.

### 2.4. Diverse in Vitro Growth Characteristics

Growth characteristics were assessed by measuring doubling times (Table 4) and by live cell imaging (Figure 5 and Figure S7) with confluence-based quantification fitted onto a curve, as described in Methods. Average time to saturation was determined based on live cell imaging data and reported as the number of days required to reach >95% confluence from a starting confluence of 5–10% (Table 4).

Average doubling time varied greatly from one cell line to the next, ranging from 1.3 to 6.2 days (Table 4). Of note, the two non-HGSC cell lines TOV2414 and TOV3392D showed significantly lower doubling time than the rest of the cell lines tested (Student’s *t*-test, *p* = 0.0013) but were not significantly different when compared to each other (Student’s *t*-test, *p* = 0.12). The confluence-based proliferation curves show that TOV2414 was the fastest-growing cell line, whereas OV2085 was the slowest (Figure 5 and Figure S7). For the majority of cell lines, the confluence-based proliferation curves showed similar results to doubling time (i.e., high proliferation rate, low doubling time value), except for OV2085 and TOV3392D, whose confluence curves indicate a slower growth than their doubling time values when compared to other cell lines (Figure 5 and Figure S7, Table 4). This discrepancy can be explained by the unique growth phenotype of these two cell lines, which causes dissociation between changes in confluence and cell number. TOV3392D has a low doubling time (2.1 days) but grows very compactly and thus takes longer to achieve confluence (26.0 days) despite the increase in cell number. OV2085, on the other hand, grows upward rather than along the bottom of the culture dish, thus proliferating without accurately reflecting this change on confluence (Figure 5 and Figure S7, Table 4). Saturation density ranged from 1.3 × 10^6^ cells for the OV3291 cell line, whose cells are morphologically large, to 15.2–17.8 × 10^6^ cells for the OV2085 and TOV3392D cell line, which have a very small and compact morphology at saturation (Table 4).

**Figure 6:**
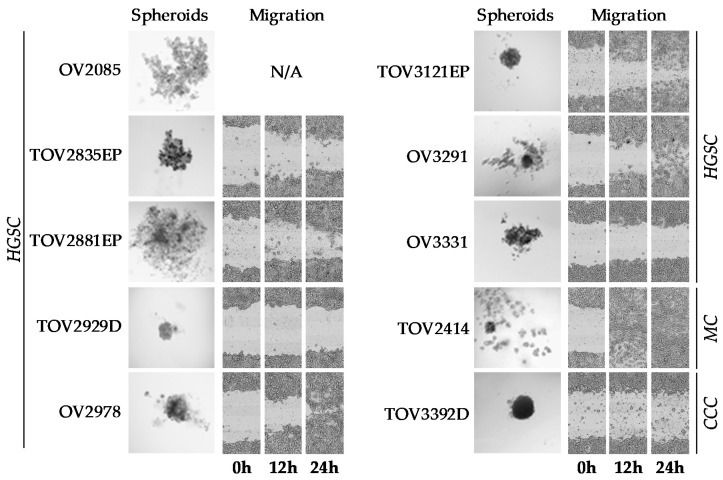
In vitro culture phenotypes. Spheroid formation of cell lines after 5–8 days using the hanging droplet technique with 2000 cells seeded, and migration evaluated by wound-healing scratch assay. Photos for migration were taken 0, 12, and 24 h after the plate was scratched. All photos are representative of three independent experiments.

As previously tested with the other EOC cell lines derived in our laboratory [39,76,77], the ability of the cell lines to form spheroids was determined using the hanging droplet method, evaluating shape, compactness, aggregation, and consistency of the spheroids formed (Figure 5, Table 4). Out of the ten cell lines tested, only TOV3392D consistently formed compact spheroids. TOV3121EP and OV3291 formed spheroids with small, compact cores surrounded by loosely aggregated cells with an irregular margin. TOV2835EP, OV2978, and OV3331 formed loose aggregates rather than solid spheroids, and TOV2929D consistently formed compact flat discs rather than spherical. OV2085, TOV2414, and TOV2881EP did not form spheroids using this method (Figure 6).

The migration potential of each cell line was evaluated by wound-healing migration assay using live cell imaging (Figure 6). Migration velocity was calculated based on wound surface closure as a function of time, as described in Methods (Table 4). TOV2414 showed the highest migration velocity (103.0 µm/h), closing the wound entirely in less than 12 h (Student’s *t*-test, *p* = 2.79 × 10^−12^), whereas TOV3392D and TOV2929D had the slowest migration velocity (0.9 µm/h and 3.8 µm/h, respectively), showing little to no ability to migrate (Student’s *t*-test, *p* = 1.64 × 10^−30^). OV3331 migrated visibly with low velocity (9.7 µm/h), whereas TOV2835EP, TOV2881EP, OV2978, TOV3121EP, and OV3291 demonstrated similar intermediate wound-healing efficacy (32.4–42.9 µm/h) (Table 4). The migration potential of OV2085 could not be determined by this assay, as this cell line could not be seeded at confluence prior to scratching, precluding the generation of a clean wound.

### 2.5. Sensitivity to Platinum-Based Chemotherapy

The carboplatin sensitivity of the cell lines was evaluated by clonogenic survival assays. Among all the cell lines tested, TOV3392D and TOV2414 were the most strongly resistant to carboplatin, with a half-maximal inhibitory concentration (IC_50_) of 18.4 and 11.2 µM respectively, significantly higher than all the HGSC cell lines (Student’s *t-*test, *p* = 8.31 × 10^−10^). This chemoresistance cannot be attributed to an inherent low growth characteristic of these cell lines, since they were found to have the fastest doubling time (Table 4). However, our results are coherent with the current knowledge of CCC and MC, which are usually intrinsically refractory to platinum-based chemotherapy [78,79,80,81]. In line with these results, patient 2414 did not respond very strongly to chemotherapy (Table 2, Figure S1). However, as opposed to what was shown in vitro, patient 3392 was surprisingly e most sensitive to chemotherapy (Table 2). This could possibly be explained by the increased efficacy of a carboplatin/paclitaxel combination, which was not tested in vitro, compared to carboplatin alone [82].

Of the other cell lines tested, OV2978 and TOV2881EP exhibited strong sensitivity to carboplatin (IC_50_ < 1 µM), significantly more sensitive than the other HGSC cell lines tested (Student’s *t-*test, *p* = 0.00054). On the other hand, TOV2835EP, TOV2929D, TOV3121EP, OV3291, and OV3331 exhibited intermediate sensitivity to carboplatin (IC_50_ between 1 and 5 µM) (Table 4). The sensitivity of OV2085 could not be determined with this assay, as this cell line did not form clones.

### 2.6. In Vivo Growth Characteristics

**Figure 7:**
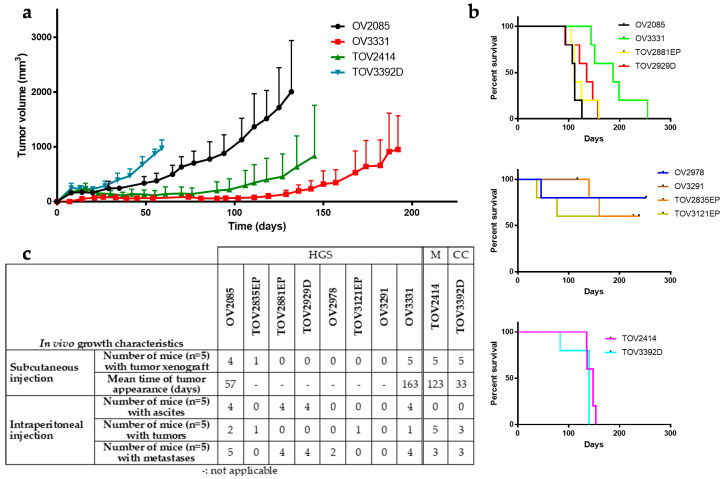
In vivo growth characteristics. (**a**) Evolution of tumor volume after SC injection in NRG mice, for cell lines that induced observable tumor growth (*n* = 5). Points represent average ± SEM, and curves were plotted when end points were attained per group, when the first animal was sacrificed. (**b**) Kaplan–Meyer survival curves of NRG mice after IP injection with each of the cell lines (*n* = 5). For clarity, cell lines were separated and grouped by HGSC that formed (**top**) or did not form (**middle**) tumors, ascites, and/or metastases in at least 3/5 mice, and non-HGSC cell lines (**bottom**). Censored data points represent mice that had reached end points. (**c**) Summarizing table of in vivo growth characteristics.

To evaluate growth potential in vivo, each cell line was injected subcutaneously (SC) or intraperitoneally (IP) in NOD rag gamma (NRG) mice (*n* = 5 mice per injection site for each cell line) (Figure 7 and Figure S8). Four of the cell lines, OV2085, OV3331, TOV2414, and TOV3392D, produced tumors in mice upon SC injection. TOV3392D produced the most rapidly growing tumors of the four cell lines, reaching 500 mm^3^ after an average of 35 days post-SC injection, but this cell line also induced severe cachexia in all mice injected SC (*n* = 10 mice over 2 experiments), forcing ethical interruption of the experiment and sacrifice of the mice between 53 and 68 days post-injection. Of the other tumor-forming cell lines, OV2085 grew the fastest, followed by TOV2414 and OV3331, which reached a tumor volume of 500 mm^3^ after an average 65, 125, and 170 days, respectively (Figure 7). None of the other cell lines could form tumors in this mouse model (Table S2, Figure S8).

Cell lines injected IP were tested for formation of ascites, tumors at site of injection, and metastases at abdominal organs. Only TOV2414 and TOV3392D formed peritoneal tumors at the site of injection. OV3331, OV2085, and TOV2929D formed medium to large metastases in most cases, whereas TOV2414, TOV2881EP, and TOV3392D formed very small metastases. OV3331, OV2085, TOV2881EP, and TOV2929D all formed large volumes of ascites, between 2 and 8.5 mL per mouse (Figure 7, Table S3).

## 3. Discussion

Epithelial ovarian cancer is a highly heterogeneous disease that poses critical challenges when choosing appropriate pre-clinical models relevant in translational science. On one hand, EOC is classified into five main subtypes (HGSC, LGSC, EC, CCC, and MC) with differences in morphology, etiology, pathogenesis, pathology, molecular biology, and prognosis [5,6,13,15,16,18,20,21,44]. On the other hand, the most frequent EOC subtype (HGSC) has a high rate of genomic instability, thus rendering it highly heterogeneous even within its own subtype [44,83,84,85]. Therefore, it is important to have reliable pre-clinical models for the rare EOC subtypes, but it is imperative to have a multitude of HGSC models to cover the different facets of this common but variable subtype.

Despite their limitations, tumor-derived cell lines remain one of the most versatile pre-clinical models; they can be cultured two-dimensionally, as three-dimensional spheroids, and even in vivo as xenografts. However, concerns exist on the process of adaptation to in vitro cell culture conditions, which can be reflected on the success rate of cell line generation. The 10 novel EOC cell lines described in this publication were derived in the same time frame (2004–2011) as 16 others from our laboratory [37,38,39], with a success rate of 13%, or slightly higher than what was recorded in the literature available at the time (0–10%, [86,87,88,89]). However, a recent publication demonstrated a 77% success rate of the establishment of ovarian cancer cell lines [90] through the use of a combination of new enriched culture medium and plates with positively and negatively charged plastic. The authors showed that their established cell lines retained the genomic landscape, histopathology, and molecular features of the original tumors. Results from the present work, together with those from our previous publication [39], also show that our cell lines retain the histological, genomic, and molecular characteristics of the original tumors. Therefore, albeit with a lower success rate, our established EOC cell lines have the potential to be used as reliable pre-clinical models for future investigations concerning new treatment modalities for the different EOC subtypes.

From the 10 novel patient-derived EOC cell lines described here, eight were identified as HGSC, one as MC, and one as CCC. Each cell line was successfully characterized as belonging to the subtype of the original patient sample by detailed protein biomarker (Figure 3 and Figure 4, Figures S2–S4) and mutational and gene expression landscapes (Figure 2, Table 3) analyses. Notably, all cell lines determined to be of the HGSC subtype harbored an annotated *TP53* mutation, and the two non-HGSC cell lines TOV2414 and TOV3392D did not. These data were confirmed by WB of whole cell lysates and IHC staining of the tumor of origin for each cell line, where cell lines with missense *TP53* mutations had a robust p53 overexpression, and those with frameshift/splice *TP53* mutations (null) did not express the p53 protein or mRNA. The two non-mutated cell lines, on the other hand, showed low or focal expression of p53 in IHC and moderate expression of the protein in WB. These are expected p53 phenotypes, as previously described [55]. Concordantly, p53 was shown to be functional only in the two non-HGSC cell lines.

Further confirmation of the HGSC subtype involved the protein expression profiles of WT1, PAX8, and ER, [55]; the mRNA expression of the *CDKN2A* gene (p16 protein) [71,72]; and sporadic mutations in the *FAT3*, *CDK12*, and *CSMD3* genes [13]. Most importantly, we demonstrated the clinical relevance of our HGSC cell lines, as their gene expression landscape was very similar to that of HGSC tumors from 593 patients from the TCGA cohort. Moreover, each of the eight HGSC cell lines described present distinct growth and tumorigenic properties relevant for pre-clinical models. In the past, our group has described and characterized 19 HGSC cell lines derived from patients’ ascites or ovarian tumor tissue [36,37,38,39]. In the present work, we describe three HGSC cell lines derived from omentum tissue (EP) that could prove relevant for studying the spread of the ovarian disease to the omentum, as this is an International Federation of Gynaecology and Obstetrics (FIGO) characteristic for stage IIIC [91], the stage in which all patients in this study were classified (Table 1). Of these cell lines, TOV2835EP and TOV3121EP did not form SC or IP tumors in NRG mice, and demonstrated strong and intermediate in vitro sensitivity to carboplatin treatment, respectively. However, the TOV2881EP cell line, which also demonstrated intermediate in vitro sensitivity to carboplatin treatment (Table 4), formed in vivo metastases and ascites when injected IP, though it did not form SC tumors (Figure 7). Interestingly, the time of sampling for this latter cell line coincided with a recurrent peak of CA125 levels in the patient of origin (Figure S1), which was not the case for the two former cell lines. Therefore, TOV2881EP might be an interesting model to study disease spread at recurrence. In this study, we also described a cell line derived from ovarian tissue, TOV2929D, with intermediate in vitro carboplatin sensitivity (Table 4), and the ability to form in vivo metastases and ascites when injected IP, but without forming tumors when injected SC (Figure 7). The time of sampling for this cell line also coincided with a CA125 peak in the patient of origin but not at a point of recurrence (Figure S1). Although the sensitivity of our cell lines to carboplatin was assessed by clonogenic survival, i.e., a single cell assay, we do not rule out the possibility that their sensitivity might differ if a cell population-based assay, such as proliferation, was to be used. This will be important to take into consideration in future drug sensitivity studies involving these novel cell lines.

Of the other HGSC cell lines, OV2085 and OV3331 are attractive pre-clinical models, in that they respectively show fast and slow in vivo growth when injected SC, and they both form IP tumors (Figure 7). These could serve as clinically relevant models to evaluate drug efficacy for treating tumors with distinct growth characteristics. The OV3331 cell line showed intermediate carboplatin sensitivity in vitro, but that of the OV2085 cell line could not be determined by clonogenic assay (Table 4). It would be interesting to evaluate carboplatin sensitivity using an in vivo model.

Cell lines OV2978 and OV3291 are derived from ascites, and are matched to their ovarian tumor cell lines derived from samples collected at the same time point for their corresponding patients [39]. For the majority of analyses, the matched HGSC cell lines behave similarly, notably in in vitro cell growth and carboplatin sensitivity, as well as the inability to form SC or IP tumors in mice. However, cell line OV3291 could not be immortalized after multiple attempts to reach cell passages higher than 55, similarly to one of our previously published cell lines, TOV-81D [36]. Upon reaching passage 50, cell proliferation is slowed, and from passage 53, cell morphology changes and cells become enlarged and non-proliferative. However, our laboratory had previously described a tumor-derived cell line from the same patient, TOV3291G. This cell line was shown to persist at least until passage 100 [39], making this pair of cell lines an interesting model to study cancer cell line immortalization. Another key difference between these two matched cell lines is the expression of WT1, an important marker of HGSC [92]. While the ascites-derived OV3291 cell line does not express WT1 in Western blot, its matched tumor-derived cell line TOV3291G, as well as its tumor of origin, does express the WT1 protein [39]. Nevertheless, the molecular identity of the OV3291 cell line in relation to TOV3291 was confirmed both at the DNA (mutational profile) and RNA (gene expression clustering) levels.

As for the MC cell line TOV2414, strong indicators for its ovarian mucinous subtype, aside from wild-type p53 and absence of WT1 expression, include the observed *KRAS* mutation, absence of SATB2 and focal expression of PAX8 [52,55,62]. This cell line is particularly interesting with respect to its very fast in vitro growth, strong migratory capacity, chemoresistance to carboplatin, and ability for form both SC and IP tumors. This model reflects the outcome of the patient who had the shortest overall survival of all patients in this study (Table 1). Our ovarian CCC cell line TOV3392D, also *TP53* wild-type, harbours an uncommon but recurrent *KRAS* mutation, in addition to its cell population phenotype of clear cells and hobnail nuclei, as well as expression of Napsin A and absence of ER and PR, strongly confirming its CCC subtype rather than metastatic endometrial carcinoma [56,66,93,94]. ARID1A and PIK3CA mutations are often immediately associated with the CCC subtype [19,50,51,95]. These genes are amongst the most recurrently mutated in this subtype; however, these mutations are estimated to have a frequency of 46–57% and 28–40%, respectively; thus, the absence of mutations of these genes in our TOV3392D cell line does not negate it as a CCC cell line. This cell line produced the most rapidly growing SC tumors in NRG mice and was the most resistant to carboplatin in vitro of all cell lines studied. It also formed IP tumors and metastasis, and mice injected with TOV3392D presented with severe cachexia, a clinically relevant ovarian cancer symptom.

Although several EOC cell lines were reported in the literature, a large number of these were insufficiently characterized, lacking important histological and molecular information. These include recently characterized ovarian cancer cell lines, where information regarding the tumor of origin, i.e., histology and protein biomarker analysis is not available for all the patients from which the cell lines were derived [54,90,96]. Moreover, systematic genomic and morphological analyses of a panel of the most often used ovarian cancer cell lines suggested that most of these cell lines were unlikely to originate from HGSC [34,35,97] and, thus, are inadequate models for studying this disease. Similarly, several non-HGSC cell lines failed to represent the EOC subtype as classified [15,34]. Our present work, which makes available 10 novel, diverse, and representative EOC cell lines, significantly improves the representation of EOC by its cell line models. Although eight of the ten cell lines presented here are of the same subtype, each presents a unique combination of individual characteristics, making them valuable models that can be combined with some of our previous well-characterized cell lines [36,37,38,39] to study a vast array of phenomena, in order to better understand this heterogeneous disease.

## 4. Materials and Methods

### 4.1. Patient and Sample Data

Tumor and ascites samples were obtained from patients from the Centre hospitalier de l’Université de Montréal (CHUM), Division of Gynaecologic Oncology, following informed consent. The stage was determined at the time of surgery by an on-site gynaecologic oncologist following the FIGO classification criteria [91]. Histology and tumor grade were evaluated by a gynaecologic-oncology pathologist according to FIGO recommendations [91]. The study was approved by the relevant institutional ethics committee, the Comité d’éthique de la recherche du CHUM (#2005-1893, BD 04.002–BSP).

### 4.2. Cell Line Establishment and Culture Conditions

Ten cell lines were established from samples originating from ten patients: 2085, 2414, 2835, 2881, 2929, 2978, 3121, 3291, 3331, and 3392. All cell lines were kept in low oxygen conditions at 37 °C, 7% O_2_, and 5% CO_2_ throughout the derivation process, following a previously established protocol [39]. Briefly, in the case of ovarian tumor (TOV) tissue-derived cell lines, tissue was scraped into a 100 mm plate with complete OSE medium (see below) and maintained for 40 days with weekly culture medium replacement. In the case of ovarian ascites (OV)-derived cell lines, patient ascites were centrifuged, where the cellular fraction was collected and seeded into a 100 mm plate, maintained by the same protocol as TOV cell lines. Cells were passaged at near-confluence and were considered immortal upon reaching 50 passages. Cells were maintained at 37 °C in low oxygen conditions (7% O_2_, 5% CO_2_) and grown in complete OSE medium, consisting of OSE medium (WISENT Inc., St-Bruno, QC, Canada), 10% fetal bovine serum (Gibco^®^, Thermo Fisher Scientific Inc., Waltham, MA, USA), 0.5 µg/mL amphotericin B (WISENT Inc.), and 50 µg/mL gentamycin sulfate (WISENT Inc., St-Bruno, QC, Canada) (normal culture conditions; NCC). Cells were passaged by trypsin 0.05% (WISENT Inc., St-Bruno, QC, Canada) digestion before reaching confluence, and culture medium was replaced weekly if cells were not passaged for more than seven days. All assays on these cell lines were conducted between passages 60 and 80, except in the case of OV3291, a non-immortalized cell line, which were conducted between passages 30 and 45.

### 4.3. Mutational Profiling

Libraries for whole exome sequencing (WES) were prepared from 500 ng of DNA using the NimbleGen SeqCap EZ Human Exome Library v3.0 kit (Roche NimbleGen, Inc., Madison, WI, USA), followed by paired-end sequencing on the HiSeq 4000 (Illumina, Inc., San Diego, CA, USA), according to recommended protocols, at the Centre d’expertise et de services (CES) Génome Québec (https://cesgq.com/). Alignment to the human genome NCBI37/hg19 was also performed by this facility to obtain BAM files of each of the 10 cell lines. BAM files were then uploaded into the public European Galaxy Network [98]. Variant calling in selected subtype-specific genes (*TP53, BRCA1, BRCA2, NF1, GABRA6, RB1, CSMD3, CDK12, FAT3, ARID1A, BRAF, PTEN, PIK3CA, CTNNB1, PPP2R1A, NRAS, KRAS*) was achieved using freebayes [99], SamTools & Bcftools [100], and reads were trimmed with a minimum Phred score of 30. Annotations were then added using SnpEff [101], dbNFSP [102] and Variant Effect Predictor [103]. From the annotated list of variant calls, only those referred as missense, splice variant, or frameshift with deleterious or damaging mutations were selected. Finally, the reads matched to variants in candidate genes were verified manually through Integrative Genomics Viewer (IGV) [104], and only variants with more than 80% of reads were accepted as homozygous somatic mutations presented in Table 4.

### 4.4. Gene Expression Microarray

RNA from 32 EOC cell lines, which includes 10 from this study and 22 previously characterized by our group [36,37,38,39], was extracted from cells when cell confluence reached 50–70%, using TRIzol™ Reagent (Invitrogen, Thermo Fisher Scientific Inc., Waltham, MA, USA), according to the recommended protocol. Microarray experiments were performed at the McGill University and the CES Génome Québec. Briefly, total RNA was quantified using a NanoDrop Spectrophotometer ND-1000 (NanoDrop Technologies, Inc., Wilmington, DE, USA), and its integrity was confirmed to have an RNA Integrity Number (RIN) of >9.0. using an Agilent 2100 Bioanalyzer (Agilent Technologies, Inc., Santa Clara, CA, USA). Sense-strand cDNA (ss-CDNA) was synthesized from 100 ng of total RNA, and fragmentation and labeling were performed to produce ss-cDNA with the GeneChip^®^ WT Terminal Labeling Kit (Applied Biosystems™, Thermo Fisher Scientific, Inc., Waltham, MA, USA) according to manufacturer’s instructions. Then 2.8 µg of DNA target was hybridized on Clariom™ S Assay HT, human (Affymetrix, Santa Clara, CA, USA) and processed on the GeneTitan^®^ Instrument (Thermo Fisher Scientific, Inc.) using the instrument’s Hyb-Wash-Scan automated workflow. Obtained CEL files were extracted and normalized using the Transcriptome Analysis Console (TAC) 4.0 software (Affymetrix). In this study, we performed subsequent gene expression analyses on the 10 cell lines described herein, as well as the matched TOV2978G and TOV3291G cell lines. For these 12 cell lines, unsupervised hierarchical clustering and principal component analysis (PCA) were performed using the TM4 MultiExperiment Viewer v3 software (MeV), a free, open-source tool for analyzing microarray data [105].

To demonstrate that the eight HGSC cell lines described here were clinically relevant, their microarray data (plus those from the two matched TOV2978G and TOV3291G cell lines) were compared to that of the publicly available Affymetrix U133a gene expression microarray data from 593 HGSC tumors from the TCGA cohort. The whole Affymetrix dataset was downloaded using the UCSC Xena Functional Genomics Explorer platform [53]. To harmonize our data with that of TCGA, the expression values of our microarray were converted to base 2 log. For both datasets, the mean values and their standard error of the mean (SEM) were calculated for each gene. We selected the 1000 most upregulated genes and the 1000 most downregulated genes from the TCGA dataset based on the average values and verified their expression in our microarray dataset. Following a previously described procedure [54], we then selected the 1000 most variably expressed genes in the TCGA dataset based on the highest SEM values. This list of genes was verified in our microarray dataset, and a Pearson correlation analysis was performed using the GraphPad Prism 6.0 software (GraphPad Software Inc., San Diego, CA, USA).

### 4.5. Immunohistochemistry

Tissue sections (4 μm thick) were entirely stained with automated stain kit (Table S4) (Ventana Medical Systems Inc., Oro Valley, AZ, USA). Slides were heated to 95 °C, and cell conditioning solutions (Ventana Medical Systems Inc.) #1 (cc1, cat.#950-124) or #2 (cc2, cat.#950-123) were added for set lengths of time for antigen retrieval (refer to Table S4 for cell conditioning solution and antigen retrieval time). Pre-diluted antibodies (Table S5) were added manually. All slides were incubated at 37 °C. Antigen-antibody reaction was revealed using Universal DAB detection kits (Ventana Medical System Inc.) (refer to Table S4). At the end of the experiment, counterstaining was achieved with hematoxylin and bluing reagent (Ventana Medical System Inc.). H&E staining was performed using the Varistain XY model of the Shandon Multi-Program Robotic Slide Stainer (Thermo Fisher Scientific Inc.) and following a standard H&E protocol. Tissue slide sections were scanned with a VS-110 microscope (Olympus, Center Valley, PA, USA) with a 20X objective. The OlyVIA v2.9 software (Olympus) was used for image analysis.

### 4.6. Western Blot

Cells were scraped from a petri dish with PBS at around 60–70% confluence, pelleted, and lysed with mammalian protein extraction reagent (Triton X-100 1%, Glycerol 10%, Tris-Base pH 4.7 50 mM, EDTA 2 mM, NaCl 150 mM) containing a protease and phosphatase inhibitor cocktail (Sigma-Aldrich Corporation, St. Louis, MO, USA) on ice for three times 5 min, vortexing between each incubation. Protein concentration was measured by Bradford protein assay (Bio-Rad Laboratories, Hercules, CA, USA) using the GENESYS 10S US-Vis spectrophotometer (Thermo Fisher Scientific, Waltham, MA, USA). Then 30 µg of total protein extract were loaded and migrated at 100V in Mini PROTEAN^®^ TGX™. Stain-Free 4–15% gradient Tris-glycine SDS-polyacrylamide 15-well gels (Bio-Rad Laboratories) and transferred onto 0.2 µm nitrocellulose membranes with the Trans-Blot Turbo transfer system (Bio-Rad Laboratories) using the mixed molecular weight program. Membranes were blocked with a PBS-Tween-milk solution (Tween 20 0.1%, milk 5%) and incubated with primary antibodies (Table S5) overnight at 4°C. Bound primary antibody was detected using horseradish peroxidase-conjugated secondary antibodies and Amersham™ ECL Prime Western Blotting detection reagents (GE Healthcare, Chicago, IL, USA). Chemiluminescence was imaged using the ChemiDoc MP Imaging System (Bio-Rad Laboratories, Hercules, CA, USA).

### 4.7. Cell Growth Rates and Saturation Density

Growth rates were determined by measuring doubling time, as previously described [36,37,39], as well as by following changes in cell confluence through live cell imaging. For determining the doubling time, cells were seeded in parallel in distinct plates of the same size with identical densities. After two to five days incubation in NCC, cells were detached with trypsin, resuspended in culture medium and counted using a hemocytometer. Cell counts at two different time points (final count and initial count) were compared, and doubling time was calculated using the following simplified version of an established formula [106]:

(1) $$Doubling\;time=\frac{\Delta t}{log_{2}\left(\frac{C_{2}}{C_{1}}\right)}$$

where *C_2_* and *C_1_* respectively represent the later and earlier cell counts at two distinct time points, and Δ*t* represents the elapsed time between cell counts *C_2_* and *C_1_*. Cell confluence was followed by live cell imaging using the IncuCyte^®^ ZOOM System (Essen BioScience Inc., Ann Arbor, MI, USA) and calculated using the IncuCyte^®^ ZOOM 2016B software (Essen BioScience Inc.), where specific confluence masks were created for each individual cell line based on their morphologies (Table S6). Estimated time to saturation (Table 4) was expressed as the average time required to reach saturation (>95%) from initial confluence (5–10%). Saturation density was defined as the mean number of cells on a 100 mm petri dish (Sarstedt, Nümbrecht, Germany) at confluence (>95%).

### 4.8. Spheroid Formation Assay

A spheroid assay was performed to determine which cell lines could form three-dimensional aggregating structures using a previously described method [39,76,77]. In 16 µL of complete OSE medium, 2000 cells were suspended and placed on the cover of a non-coated plastic petri dish, which was subsequently inverted. Sterile PBS was added to the bottom of the plate to prevent evaporation of the hanging droplets. Droplets were incubated in NCC for 6 to 10 days, and spheroid formation ability was classified based on shape and compactness of the three-dimensional structures.

### 4.9. Wound-Healing Assay

Cells were seeded at confluence in a 96-well plate and incubated in NCC for 24 h. Wells were subsequently scratched using the IncuCyte^®^ WoundMaker (Essen BioScience Inc., Ann Arbor, MI, USA), washed with PBS, and incubated in NCC. Scratches were monitored by live cell imaging using the IncuCyte^®^ ZOOM System (Essen BioScience Inc., Ann Arbor, MI, USA), and images were analyzed using the publicly available MRI Wound Healing Tool macro on ImageJ [107,108]. Residual scratch width was calculated by dividing the calculated area by image length, and velocity was expressed as µm/h.

### 4.10. Carboplatin Sensitivity Assay

Carboplatin sensitivity was determined by clonogenic survival assays, as previously described [38,109]. Cells were seeded in a series of 6-well plates at a cell-line dependent density which allowed the formation of isolated clones: 750 cells/well (TOV2414; OV3331), 1500 cells/well (TOV2835EP; OV3291), 2000 cells/well (TOV3121EP), or 4000 cells/well (TOV2881EP; TOV2929D; OV2978; TOV3392D). Cells were incubated in NCC for 24 h, after which the culture medium was replaced with complete OSE medium containing carboplatin (Accord Healthcare Inc., Kirkland, QC, Canada) at varying concentrations (0–100 µM). Cells were incubated with carboplatin for 24 h, after which the treatment medium was replaced by fresh complete OSE medium, and cells were then incubated until colonies were visible at a 2× magnification (6–21 days). Plates were then fixed for ten minutes with cold methanol (Chaptec Inc., Montréal, QC, Canada) and colored for ten minutes with a solution of 50% *v*/*v* methanol and 0.5% *m*/*v* methylene blue (Acros Organics, Thermo Fisher Scientific Inc., Fair Lawn, NJ, USA). Colonies were counted using a stereomicroscope, and the count for each concentration of treatment was reported as mean percent of control wells. Half maximal inhibitory concentration (IC_50_) values were calculated using the GraphPad Prism 7 software (GraphPad Software Inc., San Diego, CA, USA). Each individual experiment was performed in duplicate and repeated three times.

### 4.11. Mouse Experiments

All animal studies were approved by the Institutional Committee on Animal Protection (Comité institutionnel de protection des animaux-CIPA) protocol according to the Canadian Council on Animal Care (CCAC) (protocols C14008AMMs and C18010AMMs). Tumorigenic potential was assayed by injection of cells in NOD.Cg-*Rag1^tm1Mom^ IL2rg^tm1Wjl^*/SzJ (NOD rag gamma; NRG) mice (The Jackson Laboratory, Bar Harbor, ME) at subcutaneous left gluteal injection (SC) or intraperitoneal (IP) sites, as previously described [39]. A total volume of 200 µL was injected in each mouse, consisting of a suspension of 5 × 10^6^ cells in 100 µL of cold Dulbecco’s phosphate-buffered saline (D-PBS) (WISENT Inc., St-Bruno, QC, Canada) and 100 µL of either Matrigel^®^ Matrix (Corning Inc., Corning, NY, USA) for SC injections or D-PBS for IP injections. The mice were housed under sterile conditions in a laminar flow environment with unrestricted access to food and water. Formation of tumors, ascites, and metastases was evaluated twice a week for over 200 days. Animals were sacrificed before the mice reached certain limit points established by CIPA in accordance with guidelines by CCAC.

## 5. Conclusions

Our work reports ten novel immortalized EOC cell lines, providing well-characterized pre-clinical models for the benefit of the research, medical, and pharmaceutical communities, in order to advance therapeutic strategies for this deadly disease. Thorough analyses of protein biomarkers, somatic mutations, and gene expression demonstrate that each cell line represents critical aspects of the histological subtypes from which they were derived. These cell lines have diverse in vitro growth characteristics critical to cover the individual differences of EOC patients. Furthermore, some of the cell lines have the ability to grow as xenografts in mice, making pre-clinical in vivo experiments possible. These reliable and versatile models offer valuable tools for the study of ovarian cancer.

## Figures and Tables

**Table 1:** Clinical characteristics of patients from whom cell lines were derived.

Patient ID	Age at Diagnosis	Survival (Months)	Disease Stage	CA125 at Presentation (U/mL)	Histo-Pathology Subtype	Somatic BRCA Status	Prior Cancer History	Prior Oncologic Treatment	Progression as Cause of Death
2085	63	28		818			None		
2835	62	22		1126			None		
2881	56	50		1260			None		
2929	77	86		74	HGSC		CR		
2978	63	28		2397		Wild-	None	None	
3121	62	47	IIIC	42		type	None		Yes
3291	59	12		1631			None		
3331	72	13		3347	AC		None		
2414	63	4		1332	MC		None		
3392	42	29		275	CCC		Breast	FEC, RT TZM	

HGSC: high grade serous carcinoma; AC: adenocarcinoma; MC: mucinous carcinoma; CCC: clear cell carcinoma; CR: colorectal, FEC: 5-fluorouracil/epirubicin/cyclophosphamide; RT: radiotherapy; TZM: trastuzumab. The dashed lines indicate that the value applies to all lines.

**Table 2:** Treatment and sampling information for samples from which cell lines were derived.

Patient ID	Cancer of Origin	Cyto-Reductive Surgery, Residual Disease †	Initial Platinum Response (6 Months After First-Line) ‡	Oncologic Treatment Received Prior to Sampling	Platinum Sensitivity at Time of Sampling	Year at Sampling	Site of Sampling	Cell Line Designation
2085	Ovaries	Primary, Optimal	Sensitive	Carboplatin, Paclitaxel	Refractory	2004	Ascites	OV2085
2835	Peritoneal	Primary, Optimal	Resistant	Carboplatin, Paclitaxel	Resistant	2006	Omentum	TOV2835EP
2881	Ovary (L)	Secondary, Complete	Resistant	Carboplatin, paclitaxel, tamoxifen	Refractory	2006	Omentum	TOV2881EP
2929	Ovary (R)	Primary, Complete	Resistant	Chemonaïve	Resistant	2006	Ovary	TOV2929D
2978	Ovaries	Primary, Complete	Resistant	Chemonaïve	Resistant	2006	Ascites	OV2978
3121	Fallopian tubes	Interval, Optimal	Sensitive	Carboplatin, Paclitaxel	Refractory	2006	Omemtum	TOV3121EP
3291	Ovaries	Primary, Complete	Resistant	Chemonaïve	Resistant	2007	Ascites	OV3291
3331	Ovaries	None	Refractory	Carboplatin, paclitaxel, epothilone B	Refractory	2007	Ascites	OV3331
2414	Ovaries	Primary, sub-optimal	Resistant	Chemonaïve	Resistant	2005	Ovary	TOV2414
3392	Ovary (R)	Primary, Complete	Sensitive	5-fluorouracil*, epirubicin*, cyclo-phosphamide*, radiotherapy*, trastuzumab*	Sensitive	2007	Ovary	TOV3392D

L: left; R: right; OV: ascites; TOV: solid tumor tissue; EP: omentum; D: right ovary; †: optimal if ≤1 cm, sub-optimal if ≥1.0 cm, complete if none, ‡: As defined by the Gynecological Cancer Intergroup (GCIG), *: Received no ovarian cancer treatment prior to sampling, but had previously received oncologic treatment for breast cancer.

**Table 3:** Profiling of deleterious homozygous mutations by whole exome sequencing and mutation analysis of EOC subtype-specific candidate genes in the derived EOC cell lines.

Cell lines	Genes					
	*TP53*	*CDK12*	*FAT3*	*CSMD3*	*KRAS*	*BRCA1, BRCA2, NF1, GABRA6, RB1, ARID1A, BRAF, PTEN, PIK3CA, CTNNB1, PPP2R1A, NRAS*
**OV2085**	c.395A > G (missense, p.K132R)	N.I.	N.I.	N.I.	N.I.	N.I.
**TOV2835EP**	c.841G > T (missense, p.D281Y)	N.I.	N.I.	N.I.	N.I.	N.I.
**TOV2881EP**	c.467G > C (missense, p.R156P)	N.I.	c.286G > C (missense, p.G90A)	N.I.	N.I.	N.I.
**TOV2929D**	c.725G > A (missense, p.C242Y)	N.I.	N.I.	N.I.	N.I.	N.I.
**OV2978**	c.920-2 A > G (splice)	N.I.	N.I.	N.I.	N.I.	N.I.
**TOV3121EP**	c.641del (frameshift, p.H214fs)	N.I.	N.I.	c.1688A > G (missense, p.N563S)	N.I.	N.I.
**OV3291**	c.745A > T (missense, p.R249W)	c.3095 + 1 G > A (splice)	N.I.	N.I.	N.I.	N.I.
**OV3331**	c.630del (frameshift, p.N210fs)	N.I.	N.I.	N.I.	N.I.	N.I.
**TOV2414**	N.I.	N.I.	N.I.	N.I.	c.35G > C (missense, p.G12A)	N.I.
**TOV3392D**	N.I.	N.I.	N.I.	N.I.	c.34G > A (missense, p.G12S)	N.I.

N.I.: none identified.

**Table 4:** In vitro growth characteristics of the cell lines.

	Cell Lines	Cell Line Growth Characteristics				Spheroid Formation	Migration Velocity (µm/h) AVG ± SEM	Carboplatin IC_50_ (µm) AVG ± SEM
		Doubling Time (Days) AVG ± SEM	Time to Saturation * (Days) AVG ± SEM	Saturation Density (×10^6^ Cells) AVG ± SEM	Number of Passages to Date			
HGSC	**OV2085**	6.2 ± 2.2	36.2 ± 3.6	15.2 ± 4.0	>100	No	N/A	N/A
	**TOV2835EP**	2.4 ± 0.3	10.3 ± 1.7	6.4 ± 0.7	>100	Aggregate	35.9 ± 4.7	1.1 ± 0.5
	**TOV2881EP**	6.1 ± 1.7	17.6 ± 2.4	3.5 ± 0.2	80	No	32.4 ± 4.2	0.8 ± 0.1
	**TOV2929D**	6.0 ± 1.1	17.9 ± 2.4	9.3 ± 2.0	>100	Flat †	3.8 ± 1.3	4.0 ± 0.8
	**OV2978**	3.8 ± 0.03	13.3 ± 1.7	3.7 ± 0.9	>100	Aggregate	42.9 ± 4.9	0.5 ± 0.2
	**TOV3121EP**	3.0 ± 0.5	12.5 ± 2.1	2.8 ± 0.1	>100	Compact core	32.6 ± 11.7	4.3 ± 0.3
	**OV3291**	4.4 ± 0.6	16.9 ± 1.8	1.3 ± 0.3	55	Compact core	39.2 ± 5.7	5.0 ± 1.2
	**OV3331**	2.7 ± 0.3	16.7 ± 3.5	9.3 ± 1.9	80	Aggregate	9.7 ± 1.2	2.6 ± 0.8
MC	**TOV2414**	1.3 ± 0.2	4.2 ± 0.3	6.6 ± 0.3	>100	No	103.0 ± 9.5	11.2 ± 3.0
CCC	**TOV3392D**	2.1 ± 0.4	26.0 ± 7.1	17.8 ± 2.7	80	Compact	0.9 ± 0.4	18.4 ± 3.8

IC_50_: half maximal inhibitory concentration; N/A: data not available; *: 95% confluence, from 5–10% starting confluence, as measured by live-cell imaging and cell line-specific confluence masks; †: spheroids were flat discs.

## Supplementary Materials

The following are available online at https://www.mdpi.com/2072-6694/12/8/2222/s1, Figure S1: Patient progression and therapy graphs, Figure S2: Additional IHC stainings, Figure S3: H&E and IHC stainings of ovarian tumors from patients 2978 and 3291, and corresponding WB of the TOV2978 and TOV3291 cell lines, Figure S4: Whole Western blots of protein expression of markers in tumor cell lines, Figure S5: Gene expression of *TP53*, *CDKN1A* and *CDKN2A*. Figure S6: Characterization of WT1 expression in the OV3291 cell line, Figure S7: Confluence-based proliferation curves by live cell imaging fitted into a single graph, Figure S8: Supplementary in vivo growth characteristics, Table S1: Additional notes on patients from whom tumors were collected, Table S2: Additional observations in in vivo growth experiments (SC injections), Table S3: Additional observations in in vivo growth experiments (IP injections), Table S4: IHC conditions for staining whole ovarian tissue, Table S5: Antibodies used for IHC and Western blot, Table S6: IncuCyte ZOOM 2016B cell line-specific confluence mask parameters.
