# Supplementary material for: Modeling the Diversity of Epithelial Ovarian Cancer through Ten Novel Well Characterized Cell Lines Covering Multiple Subtypes of the Disease
Source: Cancers (Basel). 2020 Aug 8;12(8):2222. doi: 10.3390/cancers12082222 (PMC7465288; doi:10.3390/cancers12082222)
Supplement: Supplementary file 1 [file cancers-12-02222-s001.pdf]

cancers

MDPI

Supplementary Materials

Modeling the diversity of epithelial ovarian cancer through ten novel well characterized cell lines covering multiple subtypes of the disease

Alexandre Sauriol, Kayla Simeone, Lise Portelance, Liliane Meunier, Kim Leclerc-Desaulniers, Manon de Ladurantaye, Meriem Chergui, Jennifer Kendall-Dupont, Kurosh Rahimi, Euridice Carmona, Diane M. Provencher and Anne-Marie Mes-Masson

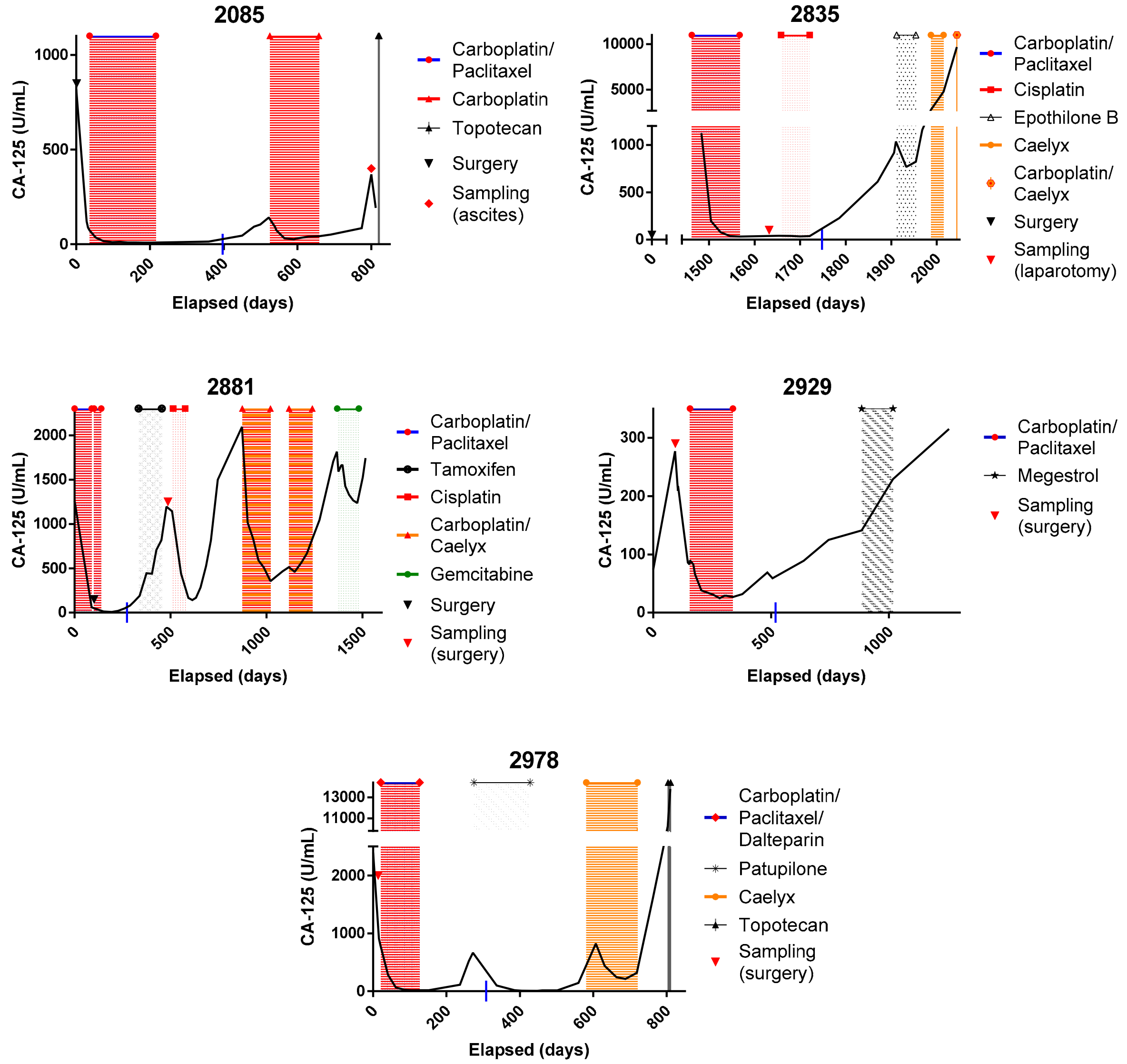

Figure S1. Patient progression and therapy graphs. Graphical representation of CA-125 levels over time. Treatment regimens are represented by highlighted zones overlapping CA-125 curves, and by color-coded symbols at the top of each graph as per graph legends. Arrowheads on the CA-125 curve indicate surgeries. The red symbol on each curve represents the sample that resulted in the patient's corresponding cell line, either arrowheads for surgeries, or diamond shapes for ascites collection. The vertical blue line on the x-axis indicates 6 months after the end of the patient's first treatment regimen.

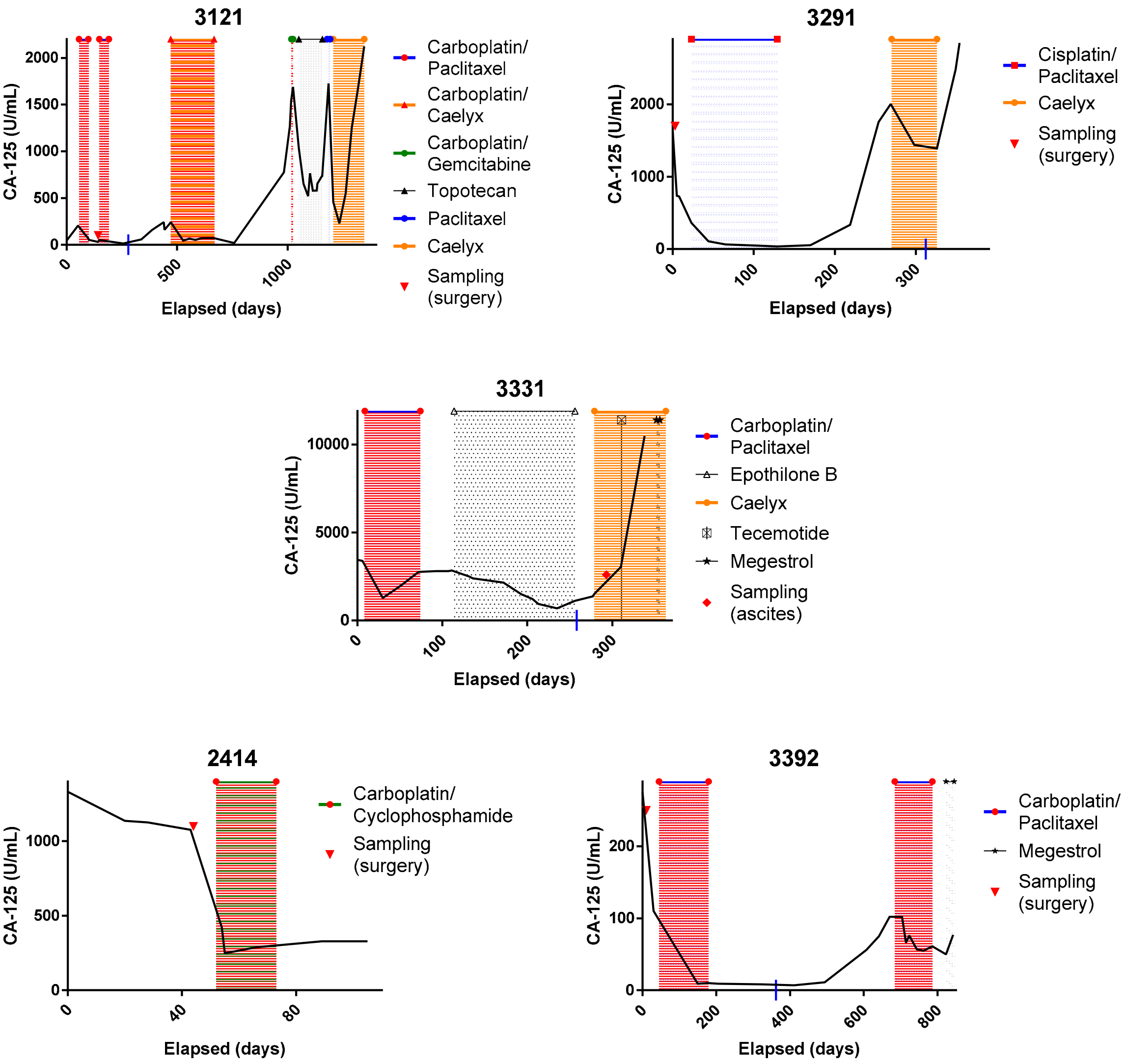

Figure S1. continued

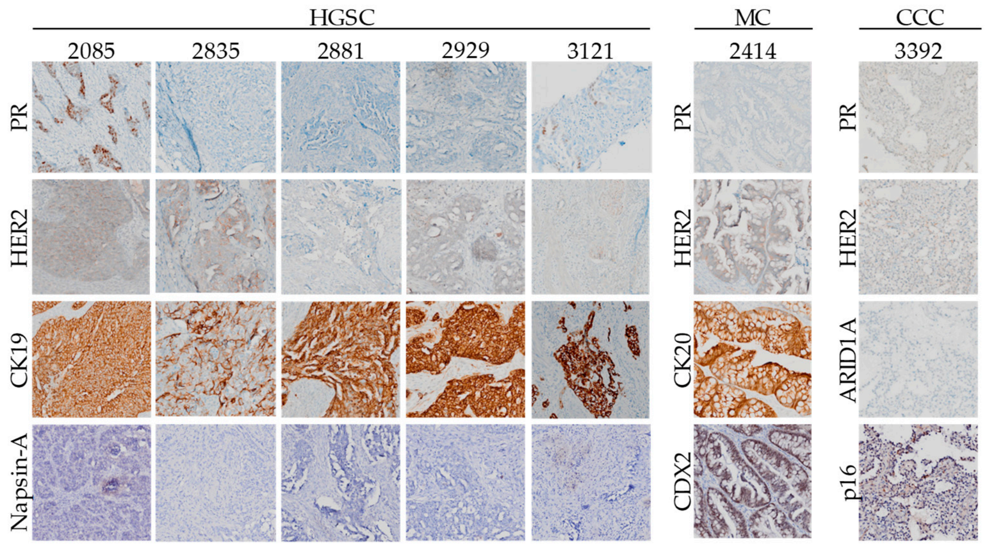

Figure S2. Additional IHC stainings. Shown are supplementary IHC staining of the tumor of origin from which each cell line was derived, separated by subtype. Each tumor was tested for relevant biomarkers for its respective subtype.

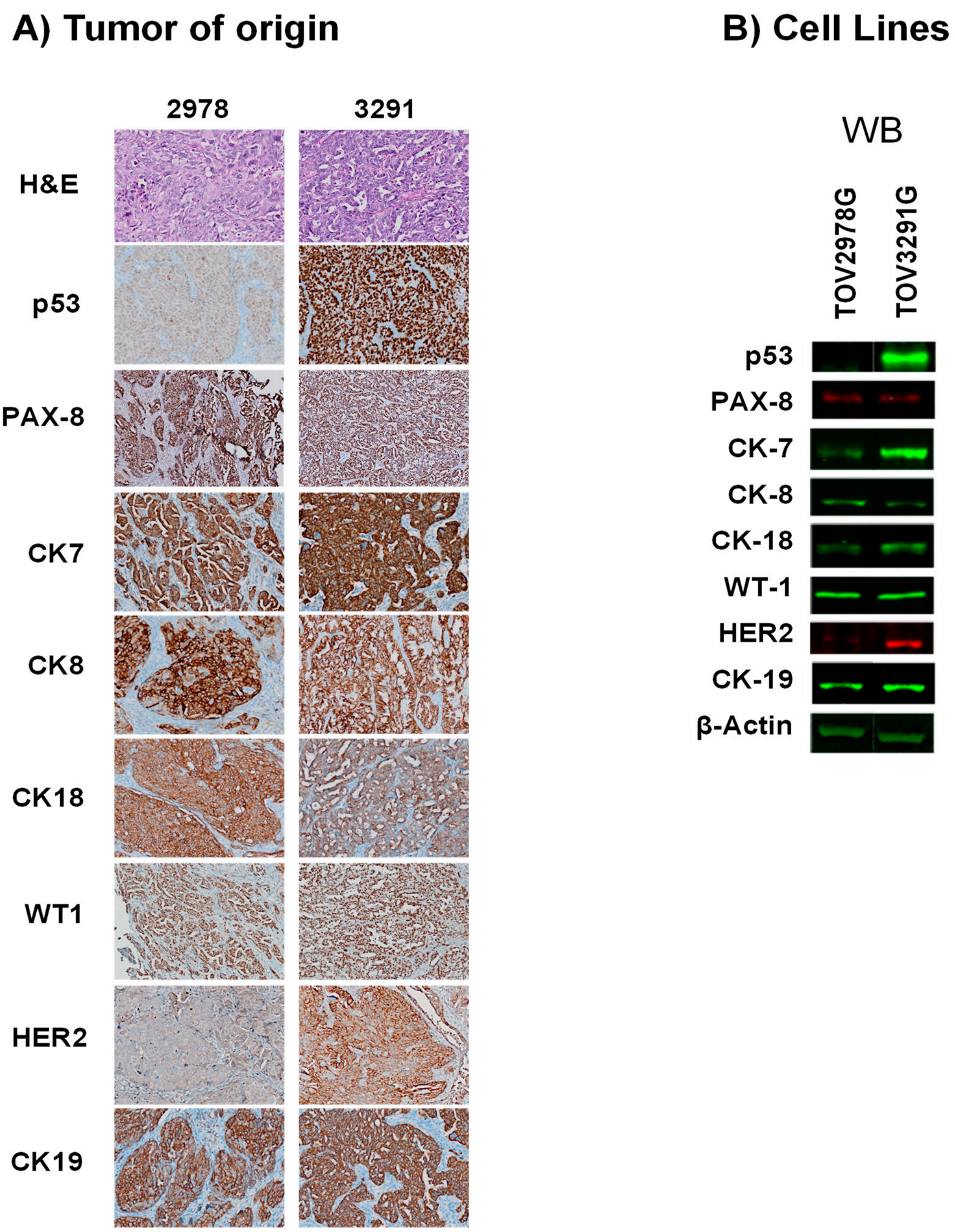

Figure S3. H&E and IHC stainings of ovarian tumors from patients 2978 and 3291, and corresponding WB of the TOV2978 and TOV3291 cell lines. Data is reproduced, with modifications, from Figure 4 of our previous publication [1] with permission from the publisher.

a

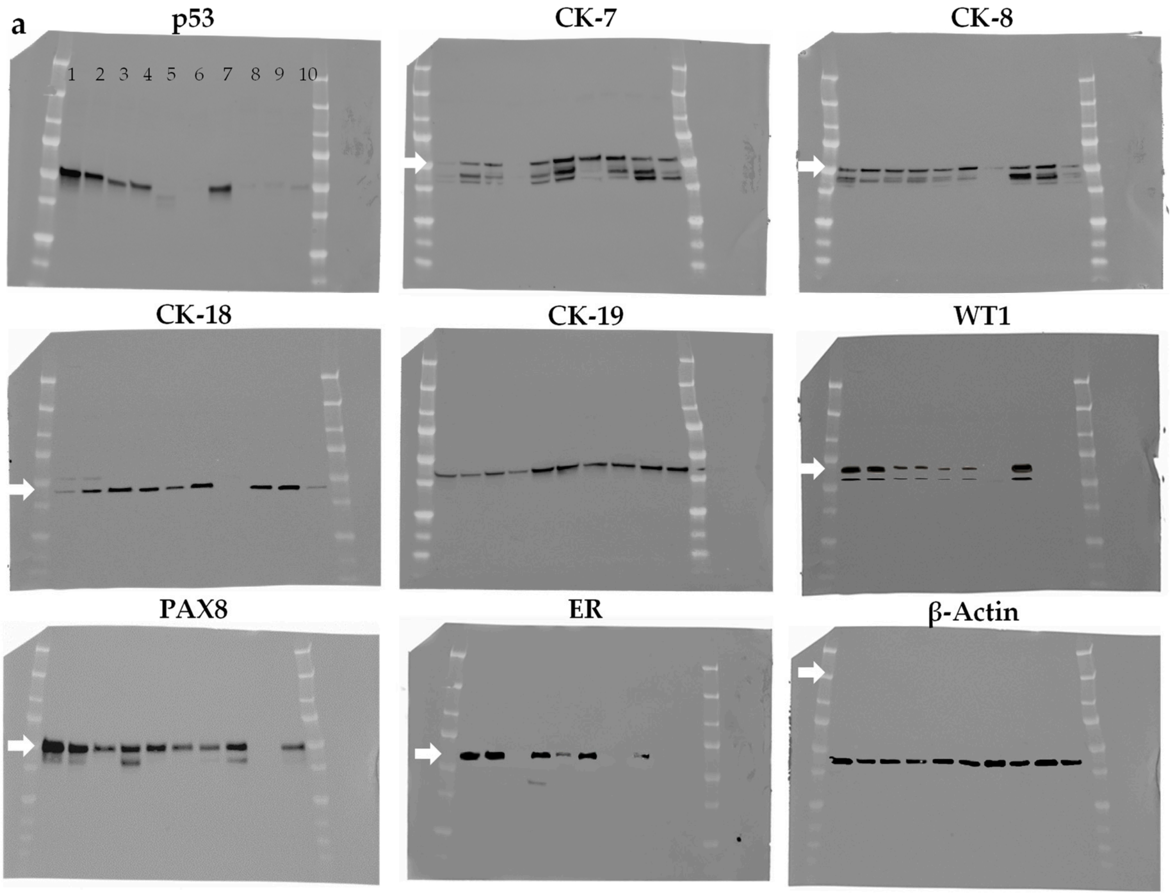

b

		p53	CK7	CK8	CK18	CK19	WT1	PAX8	ER
Lane 1	**OV2085**	1.24	0.04	0.38	0.14	0.43	1.16	1.46	1.16
Lane 2	**TOV2835EP**	0.69	0.26	0.56	0.49	0.32	1.00	0.91	1.15
Lane 3	**TOV2881EP**	0.33	0.34	0.45	0.76	0.51	0.47	0.42	0.01
Lane 4	**TOV2929D**	0.46	0.01	0.46	0.63	0.26	0.46	0.57	0.91
Lane 5	**OV2978**	0.01	0.48	0.31	0.35	1.13	0.36	0.62	0.41
Lane 6	**TOV3121EP**	0.01	0.91	0.55	0.88	0.99	0.46	0.45	0.88
Lane 7	**OV3291**	0.56	0.85	0.06	0.00	0.66	0.00	0.36	0.00
Lane 8	**OV3331**	0.02	0.81	0.53	0.71	0.67	1.37	0.70	0.42
Lane 9	**TOV2414**	0.03	0.47	0.67	0.80	0.71	0.00	0.01	0.01
Lane 10	**TOV3392D**	0.05	0.40	0.13	0.11	0.68	0.00	0.43	0.01

Figure S4. Whole Western blots of protein expression of markers in tumor cell lines. (**a**) Detection of characteristic subtype-specific EOC markers (p53, CK7, CK8, CK18, CK19, WT1, PAX8 and ER) in whole cell lysates of each cell line, with β-Actin as a control. All blots were loaded with the same sample order, indicated by lane numbers on the top left panel. Correspondence of lane numbers and cell lines is indicated in (**b**). (**b**) Intensity ratio of biomarkers for each cell line, normalized with β-Actin.

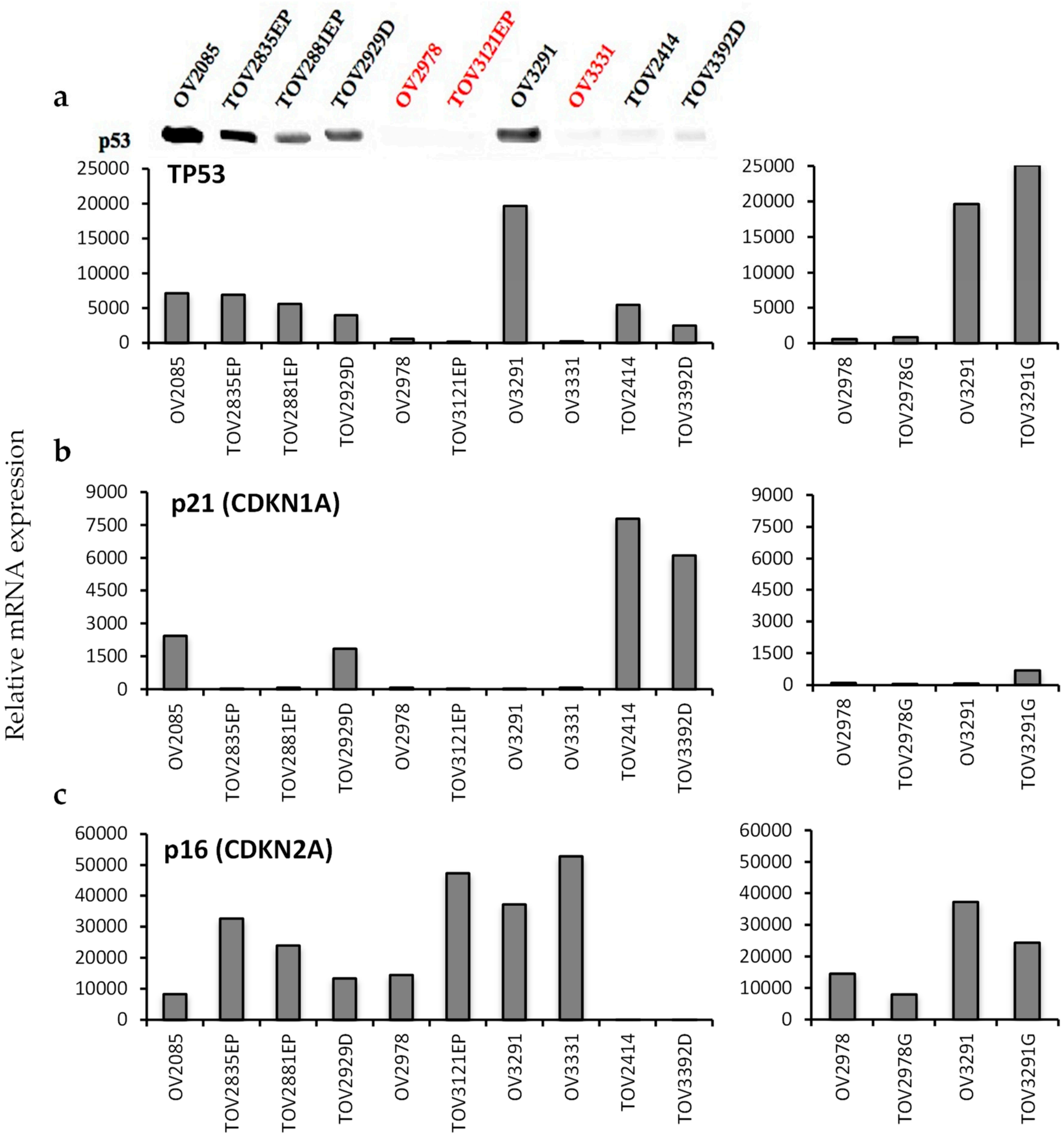

Figure 5. Gene expression of *TP53* (**a**), *CDKN1A* (**b**) and *CDKN2A* (**c**). Left panels are gene expression in the 10 novel EOC cell lines described in this work, whereas right panels are gene expression in the matched ascites and tumor cell lines derived from patients 2978 and 3291. The image on top (**a**) (left) shows protein bands of p53 from the WB of Figures 4 and S4, for ease of comparison. Red fonts denote cell lines with frameshift or splicing mutations.

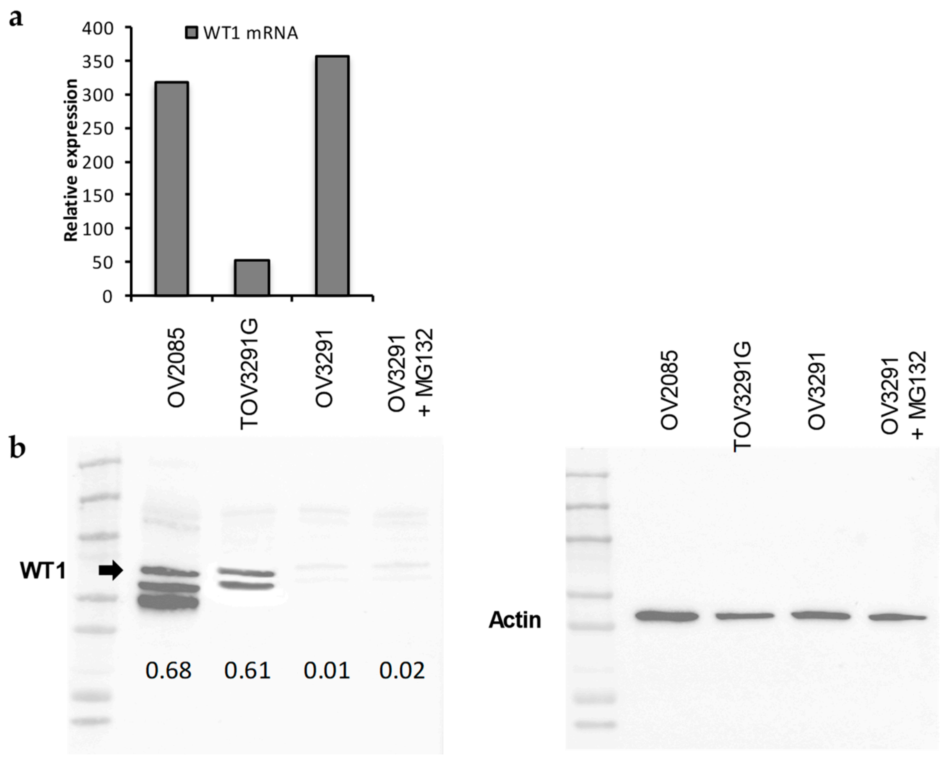

Figure S6. Characterization of WT1 expression in the OV3291 cell line. (**a**) WT1 mRNA and (**b**) WT1 protein expression was analyzed in OV2085 (as a positive control), TOV3291G and OV3291 cells. Correlation between mRNA and protein expression of WT1 was observed in OV2085 and TOV3291G, but not in OV3291. WT1 protein was also analyzed in OV3291 treated for 2 h with MG132. MG132 is a proteasome inhibitor that reduces the degradation of ubiquitin-conjugated proteins. Intensity ratio of WT1 protein for each cell line was normalized with β-Actin.

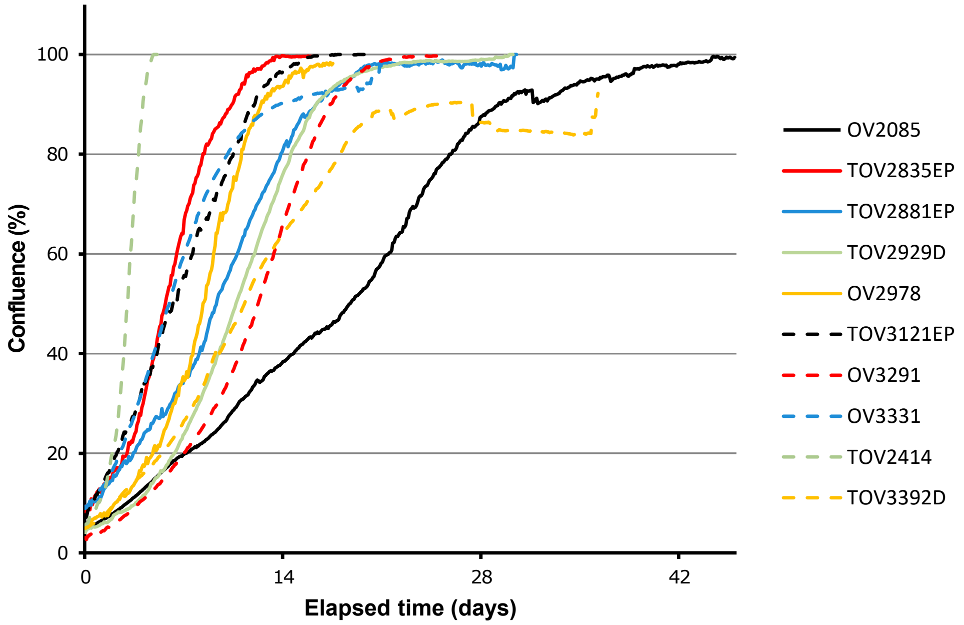

Figure S7. Confluence-based proliferation curves by live cell imaging fitted into a single graph. Cell proliferation of each cell line was determined by measuring confluence every 2 h. Initial values of confluence were between 5 and 10%, and cells were left to proliferate until confluence reached approximately 100%. Curves were fitted to a single graph for easier comparison between cell lines. Refer to Figure 4 for the SEM of each curve.

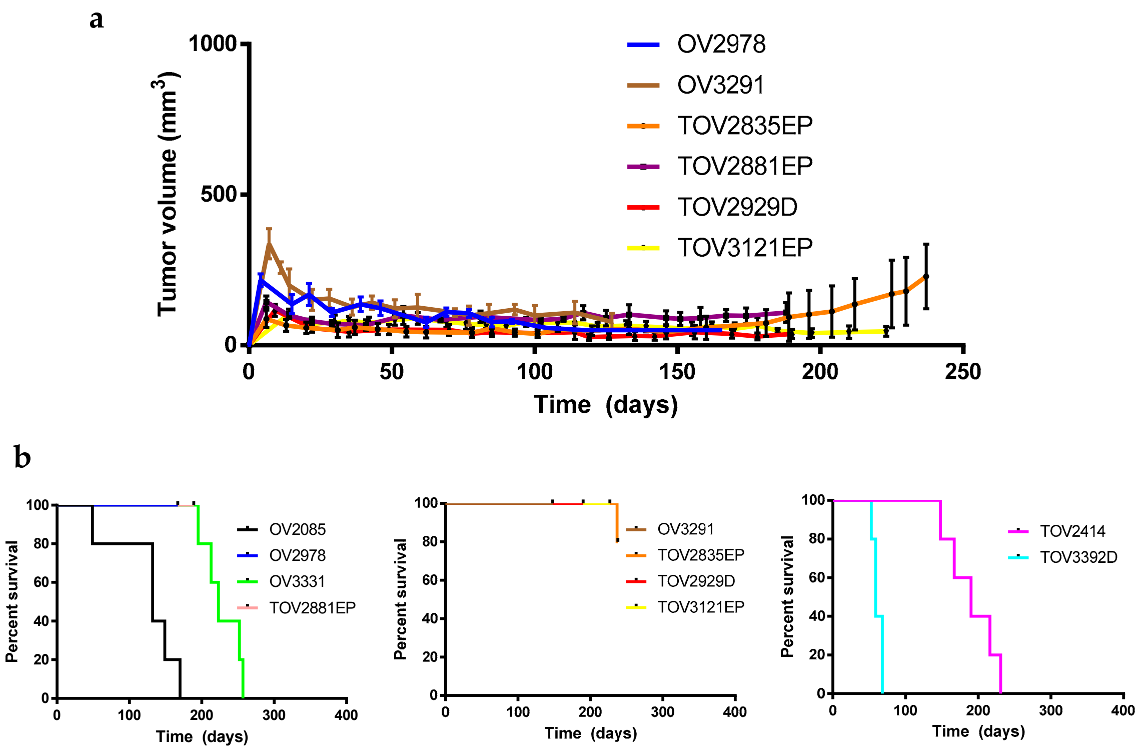

Figure S8. Supplementary in vivo growth characteristics. (**a**) Evolution of tumor volume after SC injection in NRG mice, for cell lines that did not induce observable tumor growth (n = 5). Points represent average ± SEM, and curves were plotted until end-points were attained per group, when the first animal was sacrificed. (**b**) Kaplan-Meyer survival curves of NRG mice after SC injection with each of the cell lines (n = 5). Cell lines were separated for clarity. Censored data points represent mice that had reached end-points.

Table S1. Additional notes on patients from whom tumors were collected.

Patient ID	Additional Notes
2085	Received bilateral salpingo-oophorectomy, total abdominal hysterectomy and omentectomy<br>Family history of breast cancer;<br>Participated in study OV-16 Arm 2 (carboplatin/paclitaxel);<br>Metastases at intra-abdominal lymph nodes, left iliac lymph node, liver and peritoneum;<br>Presence of ascites
2835	Received bilateral salpingo-oophorectomy and total abdominal hysterectomy<br>Patient tissue was negative for ovarian cancer at surgery in 2001, but peritoneal lavage was positive;<br>Patient only received treatment in 2005;<br>Received cisplatin intraperitoneally due to persistent miliary disease;<br>Participated in study CEPO906A2203E1 (epothilone B);<br>10% clear cells at histopathology;<br>Metastases at peritoneum, rectouterine pouch, omentum, right inguinal lymph node, right breast and liver (right lobe)
2881	Received bilateral salpingo-oophorectomy, total abdominal hysterectomy and omentectomy<br>Family history of malignant neoplasm of the urinary tract;<br>Received cisplatin intraperitoneally;<br>Metastases at pelvic and retro-peritoneal lymph nodes, sigmoid colon, peritoneum and liver
2929	Received bilateral salpingo-oophorectomy, total abdominal hysterectomy and omentectomy<br>Previous recto-sigmoid junction cancer, treated by partial colectomy;<br>Ovarian metastases at small intestine, intra-abdominal lymph nodes and right kidney;<br>Refused chemotherapy at third-line treatment, and was thus treated with Megestrol
2978	Received bilateral salpingo-oophorectomy, total abdominal hysterectomy and omentectomy<br>Participated in study CEPO906A2303 (patupilone);<br>Participated in study FOCUS Arm 1 (dalteparin);<br>Metastases at peritoneum;<br>Presence of ascites
3121	Received bilateral salpingo-oophorectomy, total abdominal hysterectomy and omentectomy<br>Metastases at peritoneum, pleural space and liver
3291	Received bilateral salpingo-oophorectomy, total abdominal hysterectomy and omentectomy<br>Carboplatin/taxol were administered both intraperitoneally and intravenously;<br>Ascites and tumor were collected at the same time;<br>Metastases at peritoneum and liver;<br>Presence of ascites
3331	Was treated in a non-affiliated hospital and was not operated, thus no histopathology subtype could be determined;<br>Death by malnutrition following disease progression;<br>Participated in study CEPO906A2203E1 (epothilone B);<br>Caelyx protocol was interrupted at patient's request;<br>Metastases at paratracheal lymph node and peritoneum;<br>Presence of ascites and pleural effusion
2414	Received bilateral salpingo-oophorectomy, total abdominal hysterectomy and partial omentectomy<br>Used Premarin for menopause between 1998 and 2001 (before diagnosis);<br>Presence of ascites
3392	Received bilateral salpingo-oophorectomy, total abdominal hysterectomy and omentectomy<br>Treated with FEC Protocol (2004-2005), radiotherapy (2005) and Trastuzumab (2005-2006) for breast cancer;<br>Ovarian metastases at peritoneum;<br>Presence of ascites

Table S2. Additional observations in in vivo growth experiments (SC injections).

Cell line	Mouse	Observations
OV2085	1117	-
	1118	-
	1125	thymic lymphoma; prostration; respiratory distress (exclusion due to early onset of thymic lymphoma)
	1127	-
	1128	-
TOV2835EP	1208	-
	1209	respiratory distress; 25% body weight loss
	1210	-
	1211	-
	1212	-
TOV2881EP	1372	-
	1373	-
	1378	-
	1380	-
	1381	-
TOV2929D	1480	-
	1481	-
	1482	-
	1483	-
	1484	-
OV2978	1335	-
	1336	-
	1357	-
	1351	-
	1352	-
TOV3121EP	1172	lots of abdominal fat
	1173	air in the intestines and fallopian tubes
	1174	-
	1175	air and fluid in fallopian tubes; pale lungs
	1176	-
OV3291	1564	-
	1565	-
	1566	-
	1572	-
	1573	-
OV3331	1087	respiratory distress; weight loss; dehydration; white mucosae
	1088	-
	1089	-
	1090	respiratory distress; thymic lymphoma; enlarged spleen; filamentous mass below the heart
	1096	-
TOV2414	1055	tumor ulceration
	1065	-
	1066	tumor ulceration; large intestinal tumor; small metastases; swollen abdomen and stomach
	1067	tumor ulceration
	1068	tumor ulceration
TOV3392D	1591	20% body weight loss
	1592	20% body weight loss
	1581	20% body weight loss
	1582	25% body weight loss; dark pink lungs
	1593	20% body weight loss
	2444	20% body weight loss
	2445	20% body weight loss; pale organs; very swollen stomach
	2446	20% body weight loss; large amount of feces in intestines
	2447	20% body weight loss; pale organs
	2448	20% body weight loss; swollen stomach

Table S3. Additional observations in in vivo growth experiments (IP injections).

Cell line	Mouse	Observations
OV2085	1129	red ascites; metastases in fat near ovaries, bladder and above liver
	1137	2.5 mL red ascites; large ovarian tumors (R + L); metastases at mesentery and between liver and stomach; enlarged spleen with white spots; pale lungs
	1138	7 mL red ascites; large metastases at bladder, liver, spleen; small metastases at stomach and intestine; swollen and red fluid-filled ovary (L); enlarged spleen; pale lungs and liver
	1139	5.5 mL red ascites; medium metastases at bladder, liver, stomach, spleen, intestines; small metastases near spleen, liver, stomach; tumors at ovaries; enlarged spleen
	1147	3 mL red ascites; inguinal and ovarian tumors; medium metastases at bladder and below stomach; small metastases near spleen, liver, stomach; enlarged spleen
TOV2835EP	1218	weight loss; enlarged belly and spleen; prostration; hard yellow mesenteric tumor; pale lungs and liver
	1219	dyspnea; body weight loss; dizzy; air in stomach, intestines, caecum; pale lungs and liver
	1220	swollen and liquid-filled red fallopian tubes
	1221	swollen and liquid-filled red fallopian tubes
	1222	small white spot on spleen
TOV2881EP	1392	2 mL pinkish brown ascites; metastases at pancreas, liver, stomach, diaphragm; small nodules on kidneys; very pale liver
	1393	2 mL red ascites; small metastases at mesentery and fallopian tubes; enlarged spleen; pale lungs
	1394	5 mL red ascites; small metastases at liver, stomach; enlarged spleen; pale liver and lungs
	1395	5 mL red ascites; small metastases at liver, stomach; enlarged spleen; pale liver and lungs
	1396	enlarged intestine
TOV2929D	1499	2 mL ascites; enlarged intestine and spleen; medium metastasis between liver and stomach; small metastases at mesentery and stomach; swollen fallopian tubes
	1500	7 mL dark red ascites; small metastases at stomach, spleen, liver; enlarged spleen; light brown liver
	1501	5 mL dark red ascites; small metastases at bladder, liver, spleen and stomach
	1502	6.5 mL dark red ascites; medium metastases at stomach; small metastases at stomach and pancreas; enlarged spleen; pale liver
	1503	body weight loss; prostration; air in intestine and fallopian tubes; fluid in ovary (L)
OV2978	1341	small metastases below stomach/liver; fat at ovaries and fallopian tubes; small pockets in fallopian tubes
	1342	enlarged red fallopian tubes and intestine
	1343	lots of fat at ovaries and fallopian tubes; enlarged red fallopian tubes; speckled liver
	1344	lots of fat at ovaries and fallopian tubes; enlarged red fallopian tubes and intestine
	1345	dyspnea; clear ascites near organs; pale and speckled lungs; small metastasis near stomach
TOV3121EP	1182	swollen abdomen; enlarged and fluid-filled gallbladder
	1183	lots of abdominal fat
	1184	lots of abdominal fat
	1185	vivid red and partly clotted ascites; lots of abdominal fat; pale kidneys and liver
	1186	large tumor at ovary (R)
OV3291	1576	internal bleeding; small metastases at mesentery near stomach
	1577	small metastasis at pancreas
	1578	-
	1579	-
	1580	-
OV3331	1097	8.5 mL red ascites; stomach and liver metastases; large metastatic tumor at ovary and fallopian tube (L)
	1098	8.5 mL red ascites; metastases at stomach, liver, ovaries, fallopian tubes; white lungs
	1106	-
	1107	6 mL red ascites; small metastases at ovaries, bladder, fallopian tubes, mesentery; swollen fallopian tubes and spleen; pale lungs
	1108	4 mL red ascites; small metastases at bladder, fallopian tubes, mesentery; pale and speckled lungs
TOV2414	1076	important body weight loss; prostration; small inguinal tumor; pale lungs; small stomach
	1078	large SC/IP tumor; 4 small mesenteric metastases near pancreas
	1081	large tumor at injection site; tumor ulceration; SC/IP tumor
	1082	medium tumor at injection site; tumor ulceration; SC/IP tumor
	1083	large SC/IP tumor; tumor ulceration; small mesenteric metastases
TOV3392D	1575	tumor at injection site and ovary (L); inguinal metastasis; small metastases at stomach and liver
	1587	enlarged fallopian tubes
	1588	SC/IP tumor at injection site; small stomach and spleen; reddish intestines
	1589	large and medium inguinal metastases; SC/IP tumor at injection site
	1590	2 inguinal metastases; small inguinal metastases

Table S4. IHC conditions for staining whole ovarian tissue.

Antibody	Antigen Retrieval	Antigen retrieval Incubation (min)	Antibody Incubation (min)	Ventana Kit	Ventana Type
ARID1A	cc1	60	60	iVIEW DAB Detection Kit	BenchMark XT
CDX2	cc1	52	24	UltraView Universal DAB Detection Kit	BenchMark ULTRA
CK18	cc1	30	40	UltraView Universal DAB Detection Kit	BenchMark XT
CK19	cc1	60	40	UltraView Universal DAB Detection Kit	BenchMark XT
CK20	cc1	60	44	UltraView Universal DAB Detection Kit	BenchMark XT
CK7	cc1	60	44	UltraView Universal DAB Detection Kit	BenchMark XT
CK8	cc1	60	60	UltraView Universal DAB Detection Kit	BenchMark XT
ER	cc1	60	60	UltraView Universal DAB Detection Kit	BenchMark XT
HER2	cc1	60	60	UltraView Universal DAB Detection Kit	BenchMark XT
MUC2	cc1	36	32	UltraView Universal DAB Detection Kit	BenchMark ULTRA
MUC5A	cc1	36	32	UltraView Universal DAB Detection Kit	BenchMark ULTRA
Napsin A	cc1	32	32	OptiView DAB Detection Kit	BenchMark ULTRA
p53	cc2	60	32	UltraView Universal DAB Detection Kit	BenchMark XT
PAX8	cc1	36	32	UltraView Universal DAB Detection Kit	BenchMark XT
PR	cc1	60	60	UltraView Universal DAB Detection Kit	BenchMark XT
SATB2	cc1	64	32	UltraView Universal DAB Detection Kit with amplification	BenchMark ULTRA
WT1	cc1	60	44	UltraView Universal DAB Detection Kit	BenchMark XT

cc1/cc2: cell conditioning #1/#2.

Table S5. Antibodies used for IHC and Western blot.

Antibody	Clone	Dilution (IHC)	Dilution (WB)	Catalog Number	Supplier	
**ARID1A**	polyclonal	1/50	-	HPA005456	MilliporeSigma	Burlington, MA, USA
CDX2	EPR2764Y	Pre-diluted	-	235R-16	Cell Marque	Rocklin, CA, USA
CK18	CD-10	1/1000	1/2000	sc-6259	Santa Cruz Biotechnology Inc.	Dallas, TX, USA
CK19	Ks19.1	1/2000	1/500	MS-198-P	Thermo Fisher Scientific Inc.	Waltham, MA, USA
CK20	SPM140	1/200	-	sc-56522	Santa Cruz Biotechnology Inc.	Dallas, TX, USA
CK7	OV-TL 12/30	1/200	1/1000	MS-1352-P	Thermo Fisher Scientific Inc.	Waltham, MA, USA
CK8	TS1	1/1000	1/500	MS-997-P	Thermo Fisher Scientific Inc.	Waltham, MA, USA
ER	SP1	1/100	1/100	ab16660	Abcam	Cambridge, UK
HER2	3B5	1/1000	1/1000	OP15L	MilliporeSigma	Burlington, MA, USA
MUC2	MRQ-18	1/100	-	291M-16	Cell Marque	Rocklin, CA, USA
MUC5A	MRQ-19	1/250	-	292M-96	Cell Marque	Rocklin, CA, USA
Napsin A	IP64	1/100	-	NAPSINA-L-CE	Leica Biosystems	Wetzlar, Germany
p53	DO-1	1/1000	1/200	sc-126	Santa Cruz Biotechnology Inc.	Dallas, TX, USA
PAX8	polyclonal	1/300	1/5000	10336-1-AP	Proteintech	Rosemont, IL, USA
PR	Y85	Pre-diluted	-	323R-17	Cell Marque	Rocklin, CA, USA
SATB2	EPNCIR130A	1/100	-	ab92446	Abcam	Cambridge, UK
WT1	6F-H2	Pre-diluted	1/250	05-753	MilliporeSigma	Burlington, MA, USA
β-Actin	AC-15		1/1000	ab6276	Abcam	Cambridge, UK
Gt x Ms	polyclonal (2°)		1/5000	AP124P	Sigma-Aldrich	St. Louis, MO, USA
Gt x Rb	polyclonal (2°)		1/10000	AP156P	Sigma-Aldrich	St. Louis, MO, USA

2°: secondary.

Table S6. IncuCyte ZOOM 2016B cell line-specific confluence mask parameters.

Parameters		HGS								M	CC
		**OV2085**	**TOV2835EP**	**TOV2881EP**	**TOV2929D**	**OV2978**	**TOV3121EP**	**OV3291**	**OV3331**	**TOV2414**	**TOV3392D**
**Segmentation augmentation**		0.5	0.5	2	1.4	1	2	1.8	1	0.7	0.1
**Cleanup**	**Hole fill (μm²)**	0	5×10^3	8×10^3	1×10^4	2×10^4	5×10^3	2×10^3	2×10^3	5×10^4	2×10^3
	**Adjust size (pixels)**	−2	8	0	−3	0	4	5	1	5	−2
**Filters**	**Area min. (μm²)**	100	1500	600	300	200	1000	850	500	1000	100
	**Area max. (μm²)**	-	-	-	-	-	-	-	-	-	-
	**Eccentricity min. (μm²)**	-	-	-	-	-	-	-	-	-	-
	**Eccentricity max. (μm²)**	-	-	-	-	-	-	-	-	-	-

References

1. Fleury, H., et al., Novel high-grade serous epithelial ovarian cancer cell lines that reflect the molecular diversity of both the sporadic and hereditary disease. Genes Cancer, **2015**, *6*, 378-398.

© 2020 by the authors. Licensee MDPI, Basel, Switzerland. This article is an open access article distributed under the terms and conditions of the Creative Commons Attribution (CC BY) license (http://creativecommons.org/licenses/by/4.0/).
